# Association of Ruminal Volatile Fatty Acids with Average Daily Gain in Growing Sheep and Goats: A Systematic Review and Meta-Regression Analysis

**DOI:** 10.3390/ani16213386

**Published:** 2026-10-27

**Authors:** Youtong Huang, Aihong Lao, Fuquan Yin

**Affiliations:** College of Coastal Agricultural Sciences, Guangdong Ocean University, Zhanjiang 524088, China; 13622275336@163.com (Y.H.); 15819933260@163.com (A.L.)

**Keywords:** ruminal volatile fatty acids, propionic acid, average daily gain, sheep and goats, multilevel meta-regression, leave-one-study-out cross-validation

## Abstract

This study assessed how ruminal volatile fatty acid (VFA) biomarkers—acetic acid, propionic acid, the acetate-to-propionate ratio (A:P), and total VFA—relate to average daily gain (ADG) in growing sheep and goats, and their added predictive value over covariates. A systematic search identified 84 unique studies (290 treatment groups; complete data: 82 studies, 283 groups). Two analytic strategies were used: inverse-variance-weighted multilevel meta-regression and an unweighted mixed model, adjusting for dry matter intake, initial body weight, crude protein, neutral detergent fiber, and species. Under inverse-variance weighting with cluster-robust inference, acetic acid (β = 0.00088; robust *p* = 0.003), propionic acid (β = 0.00188; robust *p* < 0.001), and total VFA (β = 0.00088; robust *p* < 0.001) were positively related to ADG, whereas A:P was not significant (robust *p* = 0.074) and behaved inconsistently (*p* = 0.015). The mixed linear framework gave consistent results (marginal R^2^ = 0.610–0.642). Leave-one-out cross-validation showed propionic acid gave the largest predictive gain (overall ΔCV-R^2^ = 0.124, 95% CI 0.012–0.326; RMSE reduced 10.84%). In conclusion, acetic acid, propionic acid, and total VFA are robustly associated with ADG, but their incremental explanatory power is modest; propionic acid offers the largest cross-study predictive increment, whereas A:P is unreliable.

## Simple Summary

Ruminants such as sheep and goats obtain most of their energy from the volatile fatty acids produced when microbes ferment feed in the rumen, the first stomach chamber. These acids, especially acetic acid, propionic acid, and total volatile fatty acids, are known to relate to growth, but whether measuring them helps predict weight gain beyond common factors such as feed intake, body weight, and diet composition has been unclear. We combined data from 85 published studies covering 290 feeding groups of growing sheep and goats. Higher concentrations of acetic acid, propionic acid, and total volatile fatty acids were consistently associated with higher average daily weight gain. Propionic acid added the most extra predictive value once the usual information was already taken into account, whereas the acetate-to-propionate ratio was not a reliable predictor. These findings suggest that rumen acid profiles, especially propionic acid, can help farmers and researchers estimate typical growth performance under similar feeding conditions. The gains in predictive ability were modest, however, so the results should be confirmed in new, independent studies.

## 1. Introduction

Rumen volatile fatty acids (VFAs) supply most of the energy that ruminants use, covering roughly 70–80% of metabolizable energy [1]. Rumen microbial fermentation yields acetic, propionic, and butyric acids as its principal end-products, of which acetic acid, propionic acid, and the acetate-to-propionate ratio (A:P) were the measures most often reported in the studies screened [2]. These acids do not form a single uniform energy pool; instead, the VFA profile emerges from the combined action of the microbial community, the diet, and how animals are managed. Fibrous feeds typically steer fermentation toward acetate, whereas readily fermentable starch favors propionate-producing taxa such as *Prevotella*, Succinivibrionaceae, and *Ruminococcus* spp., raising the share of propionic acid [3,4]. Propionic acid in turn is a major substrate for hepatic gluconeogenesis: it feeds the tricarboxylic acid cycle through the propionate pathway and can contribute around 60–74% of the carbon skeleton of glucose [5]. Since the propionic acid fraction reflects both fermentation type and energy partitioning, it may carry information about animal growth that other measures do not.

Average daily gain (ADG) is widely used as a growth indicator in sheep and goats [6]. Calcium propionate supplementation improved lamb growth in one meta-analysis [7], and goats with high daily gain showed higher ruminal propionate concentrations [8], as did high-yielding Hu sheep [9]. A meta-analysis of goats likewise reported that additives such as yeast, which raise ruminal propionate and total VFA, improved ADG [10]; in lambs, post-weaning ADG has been connected to rumen microbiota composition and its fermentation products [11]. Further evidence suggests that differences in the ruminal and hindgut microbiota can directly affect ADG [11].

The literature was searched systematically in line with the PRISMA guidelines [12], identifying 84 unique studies with 290 treatment groups. Complete data—all covariates plus the standard error of ADG—were available for 82 independent studies covering 283 treatment groups. Two analytical routes were then followed: inverse-variance-weighted multilevel meta-regression [13] as the primary analysis, and an unweighted mixed linear model as a confirmatory check. Both were used to examine how acetic acid, propionic acid, A:P, and total VFA relate to ADG in growing sheep and goats, to characterize their functional forms, and to adjust for feed intake, initial body weight, and diet composition. Incremental predictive value and the limits of applicability were assessed with the incremental heterogeneity-explanation proportion, species interaction tests, and leave-one-out cross-validation. Our hypothesis was that propionic acid adds predictive value for ADG beyond standard animal and dietary information, largely because of stable between-study differences in fermentation profiles that trace back to the biological mechanisms of the fermentation–gluconeogenesis axis.

## 2. Materials and Methods

### 2.1. Data Sources and Retrieval Strategy

**Figure 1:**
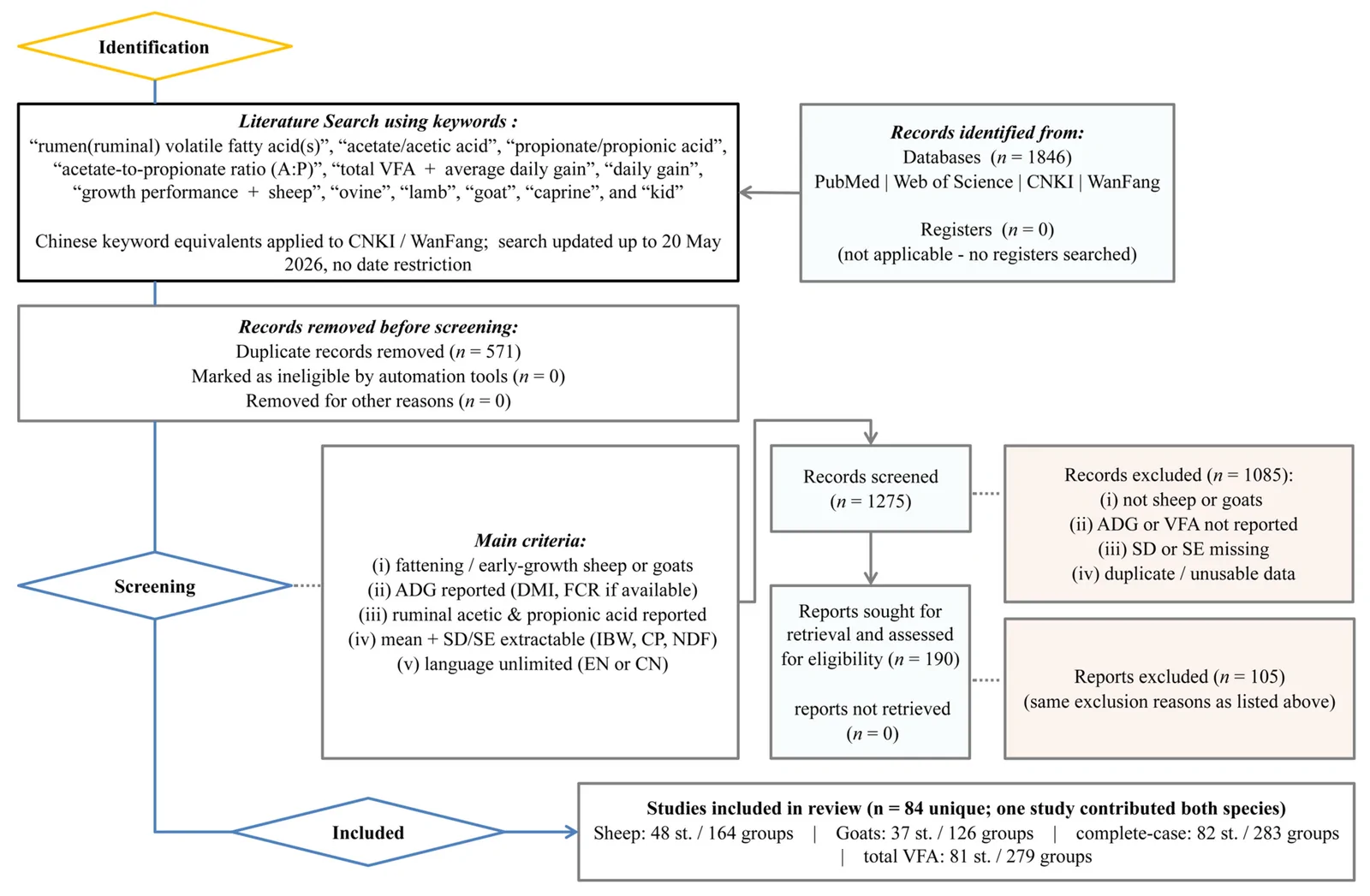
PRISMA 2020 flow diagram of the literature search and screening process. Solid arrows indicate the sequential screening process for eligible studies, while dashed branches list the excluded records and the reasons for exclusion at each stage.

The systematic literature search was conducted in accordance with the PRISMA guidelines [12] (search databases, search terms, and search date are detailed in Supplementary Text S1). No lower date restriction was applied; searches were conducted through 20 May 2026. A total of 1846 articles were identified, and after deduplication, 1275 articles remained; these underwent a preliminary screening based on titles and abstracts, eliminating 1085 articles. Among the remaining 190 articles, full-text evaluations were performed; subsequently, 105 articles were excluded due to reasons such as failure to report ADG or VFA metrics, lack of standard deviation or standard error, experimental subjects not being sheep or goats, or duplicate publications. Ultimately, 84 unique studies comprising 290 treatment groups were included (48 studies contributing 164 sheep treatment groups and 37 studies contributing 126 goat treatment groups, with one study contributing both species; the literature screening process is detailed in Supplementary Text S1 and is illustrated in Figure 1). A complete data sample was defined as records containing all predictive factors, covariates (DMI, IBW, CP, NDF), and the standard error (SE) for ADG; this resulted in 82 independent studies and 283 treatment groups, and Table 1 lists these 82 studies (full citations are provided in Table S6) (46 studies contributing 157 sheep treatment groups and 37 studies contributing 126 goat treatment groups, with one study contributing both species). For the total VFA analysis, since some studies did not report values, the analysis was based on 81 studies and 279 treatment groups. The means, standard deviations, and ranges for each predictive factor and covariate are presented in Table 2 of the article. The covariates included dry matter intake (DMI, kg/d), initial body weight (IBW, kg), dietary crude protein (CP,% DM), and neutral detergent fiber (NDF,% DM); these were independently extracted from the original manuscripts of each included study by two researchers and cross-verified; missing records were excluded according to the complete data principle. The selection of the above four factors was based on their decisive role in determining growth performance (energy intake, body size, and fiber regulation), supplemented by model comparisons incorporating stepwise addition of covariates (using AIC and heterogeneity explanation proportions) and a variance inflation factor (VIF) diagnostic (with all VIFs ≤ 2.33) to validate their necessity and ensure non-collinearity (see Table S8, covariate diagnostics). Data were analyzed using R software (version 4.5.1 (2025-06-13 ucrt); R Foundation for Statistical Computing, Vienna, Austria) within the RStudio environment (version 2025.09.1; Posit Software, PBC, Boston, MA, USA).

To enable reproducibility, every keyword was entered separately in each database and the results were screened by hand; for the English-language databases, the terms combined the VFA descriptors (rumen volatile fatty acid(s), acetate/acetic acid, propionate/propionic acid, acetate-to-propionate ratio, total VFA) with the outcome (average daily gain, daily gain, growth performance) and population (sheep, ovine, lamb, goat, caprine, kid) terms, and the corresponding Chinese-language terms (listed in Supplementary Text S1) were used for the Chinese databases (CNKI and WanFang). Because the searches were executed manually keyword by keyword, per-database hit counts and Boolean search strings were not logged (see Supplementary Text S1 for the full log). Eligibility screening was performed independently by two reviewers, first on titles and abstracts and then on full texts, with disagreements resolved by discussion until consensus was reached, and all data extracted from the included studies were cross-verified.

Because the different VFA indicators were not reported in every study, each analysis used its own complete-case sample, defined as records with all four covariates, the ADG standard error, and the indicator under study: acetic acid and propionic acid, *n* = 283 treatment groups (82 studies); the A:P ratio, *n* = 277 groups (80 studies); and total VFA, *n* = 279 groups (81 studies). The joint prediction equation presented in Section 2.2, which requires all four VFA indicators to be reported simultaneously in the same treatment group, was fitted on the stricter complete-case sample of *n* = 273 groups (79 studies). The slightly different sample sizes reported in the tables and figures therefore reflect these per-indicator missingness patterns rather than any inconsistency in the underlying dataset. During data verification, two included records were flagged as physiologically implausible (a sheep study with a total VFA of 369 mmol/L and acetate of 225.5 mmol/L, and a goat study with a dietary CP of 60.7% of DM); a sensitivity analysis excluding these two studies did not change the direction or statistical significance of any association. In addition, one publication reported both a sheep and a goat treatment arm under identical dietary treatments; it was treated as a single study, giving 84 unique studies and 82 complete-case studies.

### 2.2. Analytical Framework

**Figure 2:**
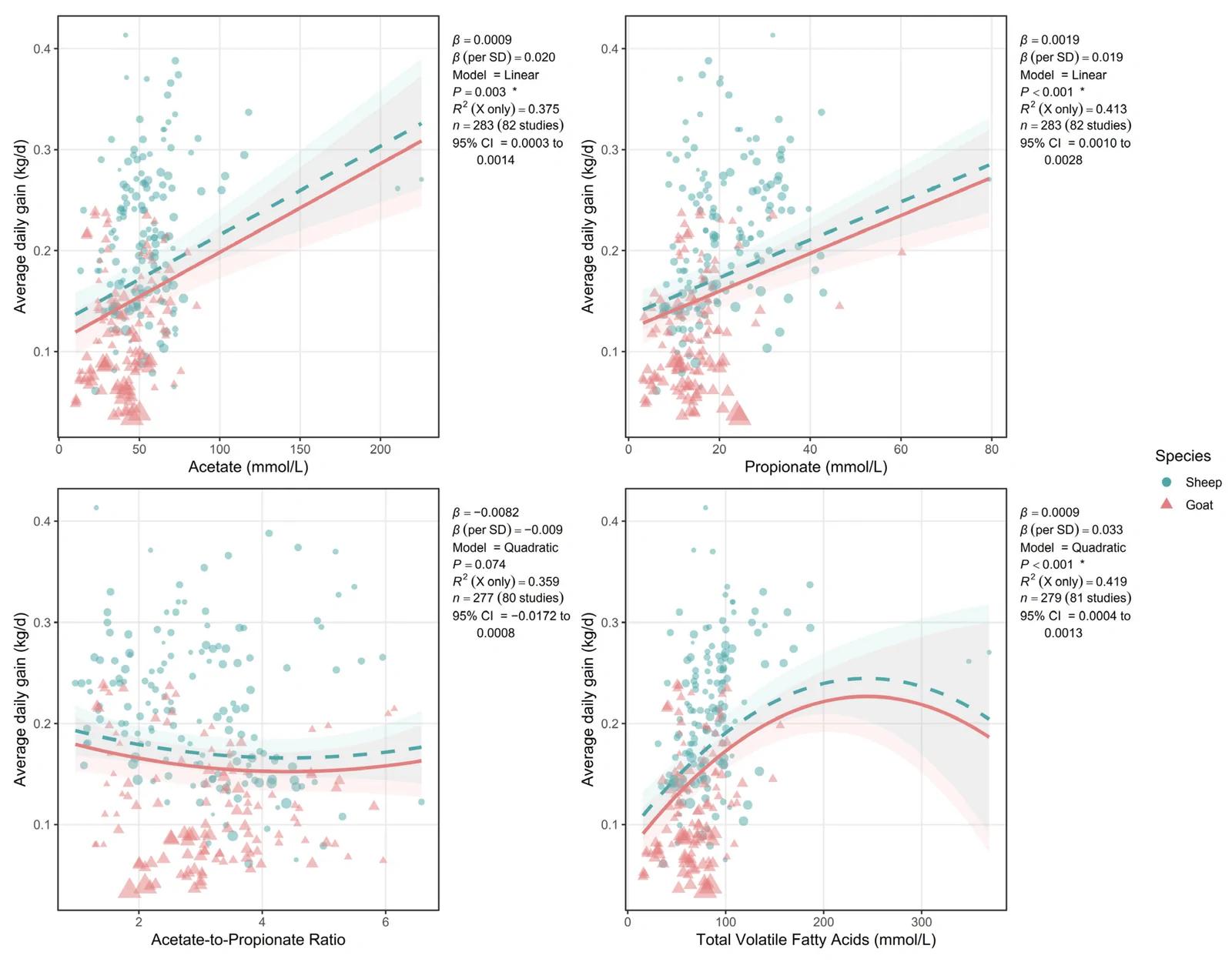
Fit of the predictive factors on ADG in inverse-variance-weighted multilayer meta-regression. Asterisks (*) indicate statistically significant differences within the same group (*p* < 0.05).

The primary analysis rested on two independent frameworks. In the first, inverse variance weighting was used: each treatment group was weighted by the reciprocal of the squared standard error (SE) of its ADG mean, and multilevel meta-regression was then applied to the group means [13]. The fitted multilevel model is given in Equation (1). Predictors were centered by their inverse-variance-weighted means, and squared terms were added to limit collinearity between X and X^2^ and to keep the main effects easy to interpret. The random-effects structure operated on two levels, study (Study) and the within-study treatment effect, the latter being the observation-level random term that captures residual between-group heterogeneity. Treating studies as clusters, we obtained cluster-robust standard errors (CR1); *p*-values under this framework are therefore cluster-robust, while within-model *p*-values are reported for reference. The second framework was an unweighted linear mixed model (LMM) in which Study entered as a random intercept and effect size (ID) was nested within Study as a random effect; marginal *R*^2^ was derived with the Nakagawa–Schielzeth method. The inverse-variance framework was designated the primary (formal) analysis and supplied the principal results, and the unweighted LMM served as a confirmatory check that the findings did not hinge on the weighting choice. Because the LMM’s random structure already accounts for within-study correlation, within-model *p*-values are reported for this framework. Heterogeneity explained is summarized by three complementary proportions (see Figure 2): the proportion for the model with predictors only (M1; the “(X only)” value in Figure 2) relative to the empty model; the incremental proportion of the full model relative to the covariates-only model (incremental *R*^2^, reported untruncated so that negative values are kept); and the proportion of the full model relative to the empty model. The output also includes *τ*^2^ for each model (spanning both study and treatment components), the residual heterogeneity Q-test, and the overall I^2^. These three measures index different benchmarks—completeness, incrementality, and overall heterogeneity—so they should not be compared directly. The covariates (DMI, IBW, CP, and NDF) were pre-specified as potential confounders on the basis of their a priori role in determining growth and were retained in all models regardless of the statistical significance of their individual coefficients. Because the four indicators are components of a common ruminal VFA pool, they are strongly interrelated (pairwise Pearson r from −0.49 to 0.91; variance inflation factors 4.3–11.0 when each indicator was regressed on the other three), and a joint inverse-variance model entering all four indicators simultaneously left none of them individually significant. The four indicators were therefore modelled in separate univariate analyses, and their coefficients should not be interpreted as mutually adjusted effects.

(1) $$Y_{\mathrm{ij}\;}={\;A}_{0}+A_{i}+{\;A}_{1i}{\mathrm{Goat}}_{\mathrm{ij}}+{\;A}_{2i}{\mathrm{Pro}}_{\mathrm{ij}}+A_{3i}{\mathrm{DMI}}_{\mathrm{ij}}+{\;A}_{4i}{\mathrm{IBW}}_{\mathrm{ij}}+{\;A}_{5i}{\mathrm{CP}}_{\mathrm{ij}\;}+{\;A}_{6i}{\mathrm{NDF}}_{\mathrm{ij}}+{\;m}_{\mathrm{ij}}$$

where Yij is the average daily gain (ADG, kg/d) of the jth treatment group in the ith study; $A_{0}$ is the overall intercept; $A_{i}$ is the random intercept for study i (random effect); $A_{1i}$ is the species-effect coefficient, with “Goat” an indicator variable taking 1 for goats and 0 for sheep; $A_{2i}$, $A_{3i}$, $A_{4i}$, $A_{5i}$, and $A_{6i}$ are the fixed-effect coefficients for propionic acid concentration (Pro, mmol/L), dry matter intake (DMI, kg/d), initial body weight (IBW, kg), dietary crude protein (CP, % DM), and neutral detergent fiber (NDF, % DM), respectively; and m_ij_ is the residual term.

(2) ADG(kg/d)=0.0349−0.0055·Goat +0.00219·Pro +0.1320·DMI−0.00131·IBW +0.00136·CP−0.00115·NDF

For the fitted equation (Equation (2)), standard errors are shown in parentheses: intercept = 0.0349 (0.0356); Goat = −0.0055 (0.0133); Pro = 0.00219 (0.00037); DMI = 0.1320 (0.0144); IBW = −0.00131 (0.00090); CP = 0.00136 (0.00085); NDF = −0.00115 (0.00048). *n* = 273 treatment groups and 79 studies; the marginal R2 (R2m) = 0.658.

### 2.3. Automatic Selection of Function Forms

**Figure 3:**
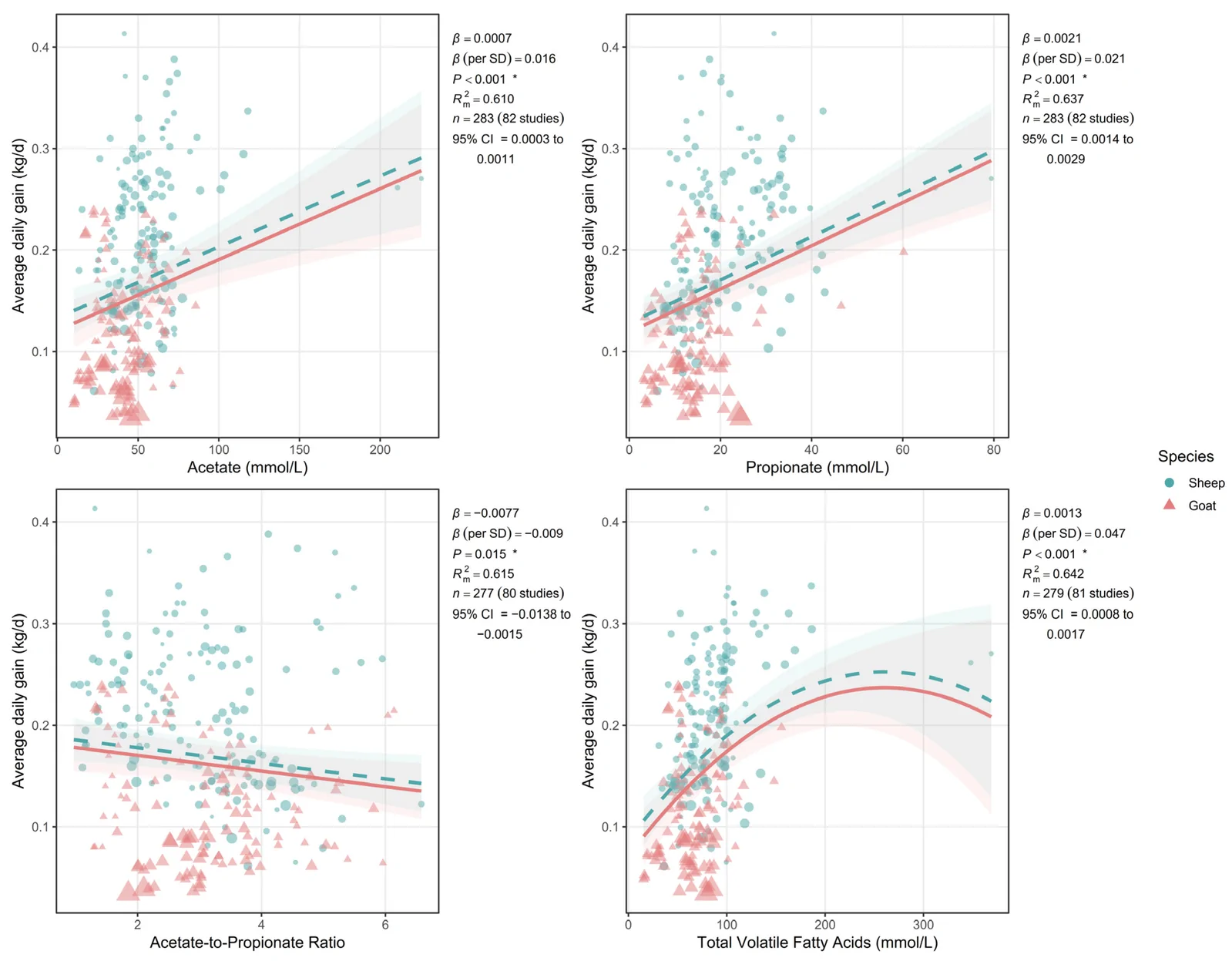
Fit of the predictive factors on ADG in an unweighted mixed linear model. Asterisks (*) indicate statistically significant differences within the same group (*p* < 0.05).

To determine whether a linear or quadratic functional form should be adopted to assess the association between each predictor (acetic acid, propionic acid, A:P, total VFA) and ADG, after controlling for covariates, linear and quadratic models were fitted separately for each indicator (with the quadratic term included as a centered variable to reduce multicollinearity); multiple pieces of evidence were then synthesized to decide on the appropriate functional form: statistical significance of the quadratic coefficient (*p* < 0.01); the incremental heterogeneity explanation proportion of the quadratic model relative to the linear model under either the inverse-variance framework (Figure 2) or the mixed linear framework (Figure 3), or the relative increase in the marginal R^2^ (≥10%); and the degree of improvement in the Akaike Information Criterion (AIC) (a decrease ≥4). The quadratic form was selected when it outperformed the linear form for an indicator; otherwise, the linear form was retained to avoid over-parameterization and extrapolation beyond observed data ranges. The results showed that, under the inverse-variance framework, the functional forms for A:P and total VFA were identified as quadratic, while those for acetic acid and propionic acid were linear; under the mixed linear framework, all indicators exhibited linear relationships.

### 2.4. Species Interaction Test

To explore the sources of heterogeneity further, the primary analysis was extended by fitting an expanded multilevel meta-regression model with average daily gain (ADG) as the outcome and species, dry matter intake (DMI), initial body weight (IBW), dietary crude protein (CP), and neutral detergent fiber (NDF) as covariates. To separate within-study from between-study sources of treatment effects, the treatment-level covariate (X) was decomposed into a within-study component (X_within_—the deviation of each treatment group’s X from the mean X of its own study) and a between-study component (Xbetween—the mean of X within each study); main effects and their species interactions were estimated separately for the two components. Weighting (inverse variance) and the random structure (studies as random intercepts, effect ID nested within each study) matched the primary analysis. For the interaction term, slope differences between sheep and goats were tested at both the within-study and between-study levels, with multiple interaction tests corrected using the BH method. When an interaction was not significant, a contrast approach estimated within-study and between-study slopes for each species with their 95% confidence intervals (CIs), and the overlap ratio among the ranges of original X, within-study X, and between-study X quantified the degree of data overlap between the two species.

### 2.5. Leave-One-Study-Out Cross-Validation (LOSO)

For each predictor, a baseline model (DMI + IBW + CP + NDF; species also included in the full sample) and a baseline-plus-indicator model were estimated. Leave-one-out cross-validation was then applied: each iteration omitted one study, refitted the model to the remaining studies, and predicted all treatment groups of the omitted study. Evaluation proceeded along three lines: overall population prediction (sheep and goats), a sheep-specific equation, and a goat-specific equation. The cross-validation *R*^2^ (CV-*R*^2^), the change in CV-*R*^2^ after adding the indicator (ΔCV-*R*^2^), the RMSE, and its percentage reduction were computed. Paired bootstrap resampling (1000 repetitions) with the study as the sampling unit supplied 95% confidence intervals (CIs) for the reductions in ΔCV-*R*^2^ and RMSE, taken as the 2.5% and 97.5% percentiles. A 95% CI for ΔCV-*R*^2^ that excludes zero together with a lower RMSE denotes clear incremental predictive value; if only the point estimate improves while the 95% CI crosses zero, predictive performance improves but the increment remains uncertain.

### 2.6. Sensitivity Analyses and Risk of Bias Assessment

The SE Monte Carlo perturbation approach drew 500 independent random values for the standard errors of the predictors, DMI, and IBW under a normality assumption, re-estimated the interaction model each time, and reported whether the perturbed percentile intervals excluded zero and whether the signs matched the primary findings. The full dataset and the complete-data subset were compared by repeatedly fitting the primary model to the entire dataset with the data-based approach, checking whether the direction, significance, and incremental *R*^2^ stayed qualitatively unchanged. Robustness was further assessed by comparing coefficients, *p*-values, and signs across four model variants: inverse variance weighting, a model with only study-level random effects, precision truncation (95th percentile), and the unweighted mixed-effects model. Publication bias was appraised with a funnel plot combined with Egger’s linear regression test, in which standardized effect sizes were regressed on the ADG standard errors of the studies to test whether the intercept deviated from zero. Egger’s test *p* values for all four indicators were <0.001, representing, at least in part, mean–variance coupling between raw group means and their standard errors rather than publication bias per se; nonetheless, selective reporting of extreme group means cannot be excluded, and association magnitudes may accordingly be inflated. These caveats should be kept in mind when interpreting them (see Table S1). Individual study risk of bias was assessed with the SYRCLE tool (Hooijmans et al., 2014), the 10-item instrument recommended for animal intervention studies (Table S7). Baseline body weight/age was reported in most studies (74 of 82) and outcome reporting was largely complete (incomplete outcome data at low risk in 80 of 82; selective reporting at low risk in 82 of 82). By contrast, sequence generation and blinding were rarely described (blinding not reported in 81 of 82) and were judged as unclear or high-risk, whereas allocation concealment, random housing, random outcome assessment and blinding of the outcome assessor were almost never described and were judged as unclear. No study was judged at high risk of bias for the ADG estimate itself. Most of the included studies were nutrition or feeding trials in which experimental groups were compared within the same herd, flock, or feeding system rather than through an independently randomized patient-level design; standard risk-of-bias instruments developed for intervention comparisons therefore have limited applicability to the group-mean data synthesized in this review. Instead, reporting bias was evaluated via funnel plots and Egger’s test, and the robustness of inferences was examined through multiple sensitivity analyses (Section 2.6). The review protocol was not registered in PROSPERO, which is restricted to systematic reviews directly relevant to human health; it was instead registered on the Open Science Framework (OSF), available at https://doi.org/10.17605/OSF.IO/J4HAR.

### 2.7. Statistical Software, Significance Definition, and Data Availability

All analyses were performed in R (version 4.5.1). Multilevel meta-regression models were fitted with the rma.mv function of the metafor package (version 4.6.0); linear mixed models (LMMs) were fitted with the nlme package (version 3.1-166) and the lme4 package (version 1.1-35.5); Satterthwaite’s approximation was obtained with the lmerTest package (version 3.1-3); data management and variable transformations relied mainly on the dplyr package (version 1.1.4). The significance level was set at α = 0.05; the association between the four VFA indicators and ADG was treated as a pre-specified primary hypothesis; *p* values were used to assess the robustness of the main effects; and multiple comparisons for species interactions were corrected with the BH method. Analysis scripts, diagnostic plots, and all intermediate results are provided in Table S8 and the Supplementary Materials.

## 3. Results

### 3.1. Sample Characteristics

The complete data sample comprised 82 studies and 283 treatment groups, with 46 studies contributing 157 sheep treatment groups and 37 studies contributing 126 goat treatment groups (one study contributed both species); the total VFA dataset included 81 studies and 279 treatment groups. The baseline characteristics and literature sources of the included studies are presented in Table 1; full citations are provided in Table S6. Within the complete data sample, the mean ADG was 0.170 ± 0.081 (SD) kg/d, the mean acetic acid was 48.30 ± 22.70 mmol/L, the mean propionic acid was 17.95 ± 9.83 mmol/L, the mean A:P was 3.06 ± 1.15, and the mean total VFA was 77.27 ± 36.77 mmol/L; the mean ADG values were 0.213 kg/d for sheep and 0.117 kg/d for goats. The means, standard deviations, and ranges of all variables are provided in Table 2. A parallel analysis using the feed conversion rate (FCR, feed intake-to-gain ratio; lower values indicate better feed efficiency) as a secondary endpoint is summarized in Section 3.9.

### 3.2. The Correlation Between Acetic Acid and ADG

Acetic acid showed a significant positive association with ADG (Table 3: *β* = 0.00088; 95% CI: 0.00031–0.00144; cluster-robust *p* = 0.003; total model heterogeneity explained = 0.583), and this relationship was also significant in the mixed linear framework (Table 4: *β* = 0.00070; 95% CI: 0.00034–0.00106; *p* < 0.001; marginal *R*^2^ = 0.610). The incremental heterogeneity explained by acetic acid in Table 3 was 0.017, the smallest among the four indicators. At the species level, sheep showed significant associations in both frameworks (Table 3: *β* = 0.00098; robust *p* = 0.013; Table 4: *β* = 0.00066; *p* = 0.004), whereas goats did not in either (Table 3: *β* = 0.00052; robust *p* = 0.205; Table 4: *β* = 0.00063; *p* = 0.065). Note, however, that the incremental heterogeneity explained by acetic acid in sheep was negative (−0.017; Table 3), indicating that the addition of acetic acid did not increase the explained heterogeneity in this subgroup (see Figure 2 and Figure 3).

### 3.3. Association Between Propionic Acid and ADG

Propionic acid showed a significant positive correlation with ADG (Table 3: *β* = 0.00188, 95% CI: 0.00095–0.00280; cluster-robust *p* < 0.001; overall model heterogeneity explanation proportion = 0.621); the mixed linear framework was also significant (Table 4: *β* = 0.00213, 95% CI: 0.00139–0.00287, *p* < 0.001; marginal *R*^2^ = 0.637). In Table 3, the incremental heterogeneity explanation proportion for propionic acid was 0.107, the highest among the four indicators. Within the species subgroup, both frameworks for goat propionic acid yielded significant results (Table 4: *β* = 0.00279, *p* < 0.001; Table 3: *β* = 0.00227, robust *p* < 0.001); for sheep propionic acid, the mixed linear framework yielded a significant result (Table 4: *β* = 0.00157, *p* = 0.002), while the inverse variance framework also yielded a significant result (Table 3: *β* = 0.00148, robust *p* = 0.035).

### 3.4. The Relationship Between A:P and ADG

The evidence strength for A:P is inconsistent across the two frameworks: in the inverse-variance framework, it does not achieve cluster-robust significance (Table 3: *β* = –0.00820; 95% CI: –0.01720 to 0.00080; robust *p* = 0.074), whereas in the mixed linear framework, it exhibits weak negative significance (Table 4: *β* = –0.00769; 95% CI: –0.01384 to –0.00153; *p* = 0.015). The incremental heterogeneity explanation proportion is 0.071 (Table 3). The contrast—where the three concentration-based indicators (acetic acid, propionic acid, and total VFA) showed positive associations while the single ratio-based indicator (A:P) showed a negative association—is consistent with the exploratory findings from the between-study decomposition analysis; however, the inconsistency in significance across the two frameworks suggests that the A:P result is not robust and should therefore not be used as an independent predictive indicator. This does not imply that A:P has no true association with ADG; rather, both frameworks consistently showed a negative, framework-inconsistent direction (Table 3 and Table 4).

### 3.5. Correlation Between Total VFA and ADG

The total VFA and ADG exhibited a significant positive correlation (Table 3: *β* = 0.00088, 95% CI: 0.00042–0.00135; cluster-robust *p* < 0.001; the incremental heterogeneity explanation proportion for total VFA reached 0.083). The mixed linear framework also showed a significant result (Table 4: *β* = 0.00127, 95% CI: 0.00080–0.00174, *p* < 0.001; marginal *R*^2^ = 0.642). At the species level, both frameworks yielded significant results for sheep (Table 3: *β* = 0.00108, robust *p* = 0.002; Table 4: *β* = 0.00157, *p* < 0.001) and for goats (Table 3: *β* = 0.00055, robust *p* = 0.033; Table 4: *β* = 0.00148, *p* = 0.039). For details, see Figure 2 and Figure 3 and Figures S1–S3.

### 3.6. Results of the Species Interaction Test

In the extended model, neither the Species × X (Within) nor the Species × X (Between) interaction term reached statistical significance (*p* > 0.05). Species-specific slopes showed that for sheep acetic acid, both the intra-study and inter-study slopes were significant (*p* = 0.005 and *p* = 0.032), whereas for goat acetic acid, neither slope was significant. For goat propionic acid, both the intra-study and inter-study slopes were significant (*p* = 0.001 and *p* = 0.031), while for sheep propionic acid, only the inter-study slope was significant (*p* = 0.008). These findings describe a point-estimate pattern in which acetic acid is the more sensitive indicator for sheep and propionic acid for goats; nevertheless, the differences were not significant enough to confirm an interaction effect.

### 3.7. Incremental Predictive Value of Leave-One-Study-Out Cross-Validation

**Figure 4:**
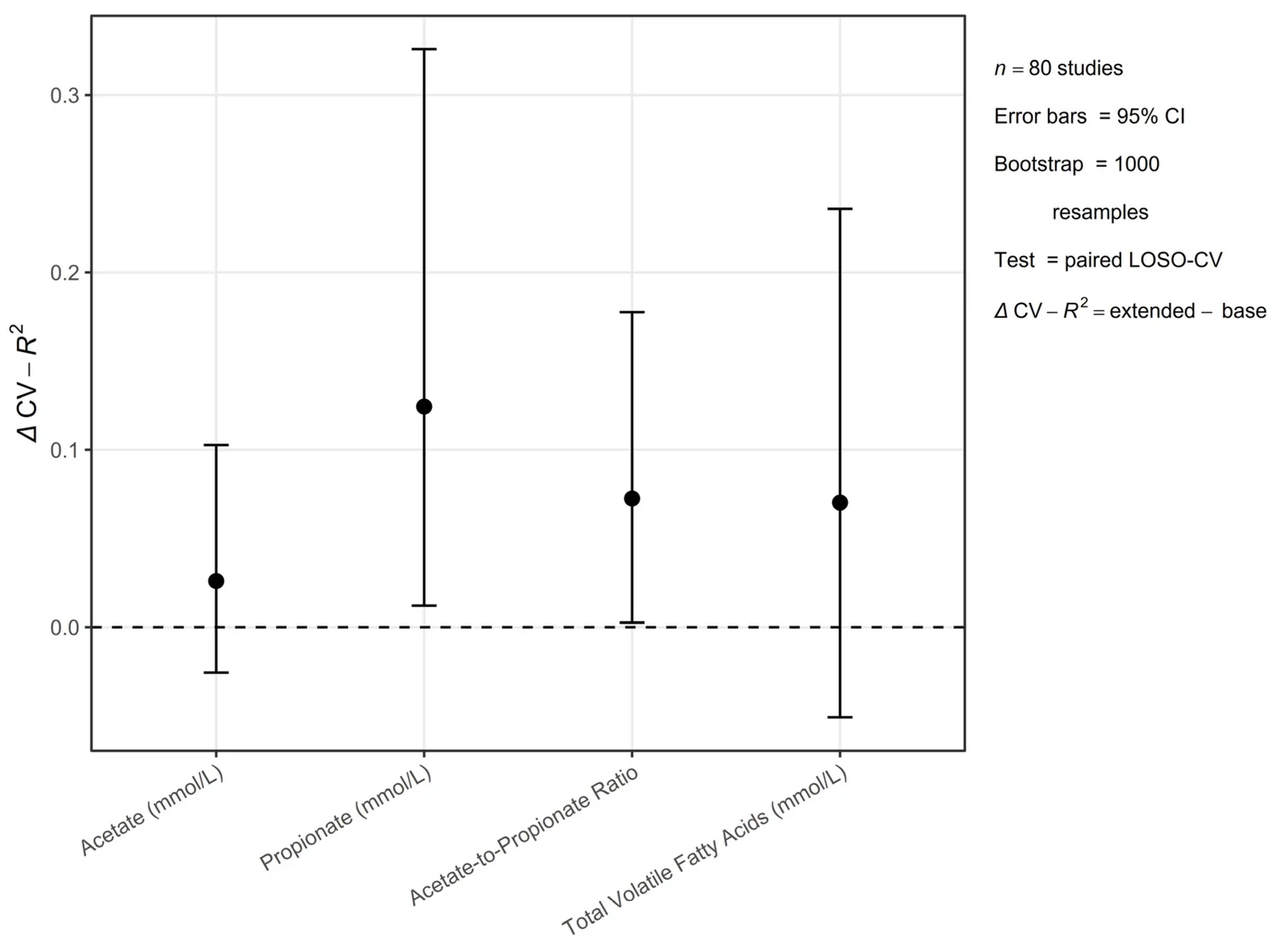
The ΔCV-*R*^2^ values for each VFA indicator along with their paired quantile 95% confidence intervals (forest map).

**Figure 5:**
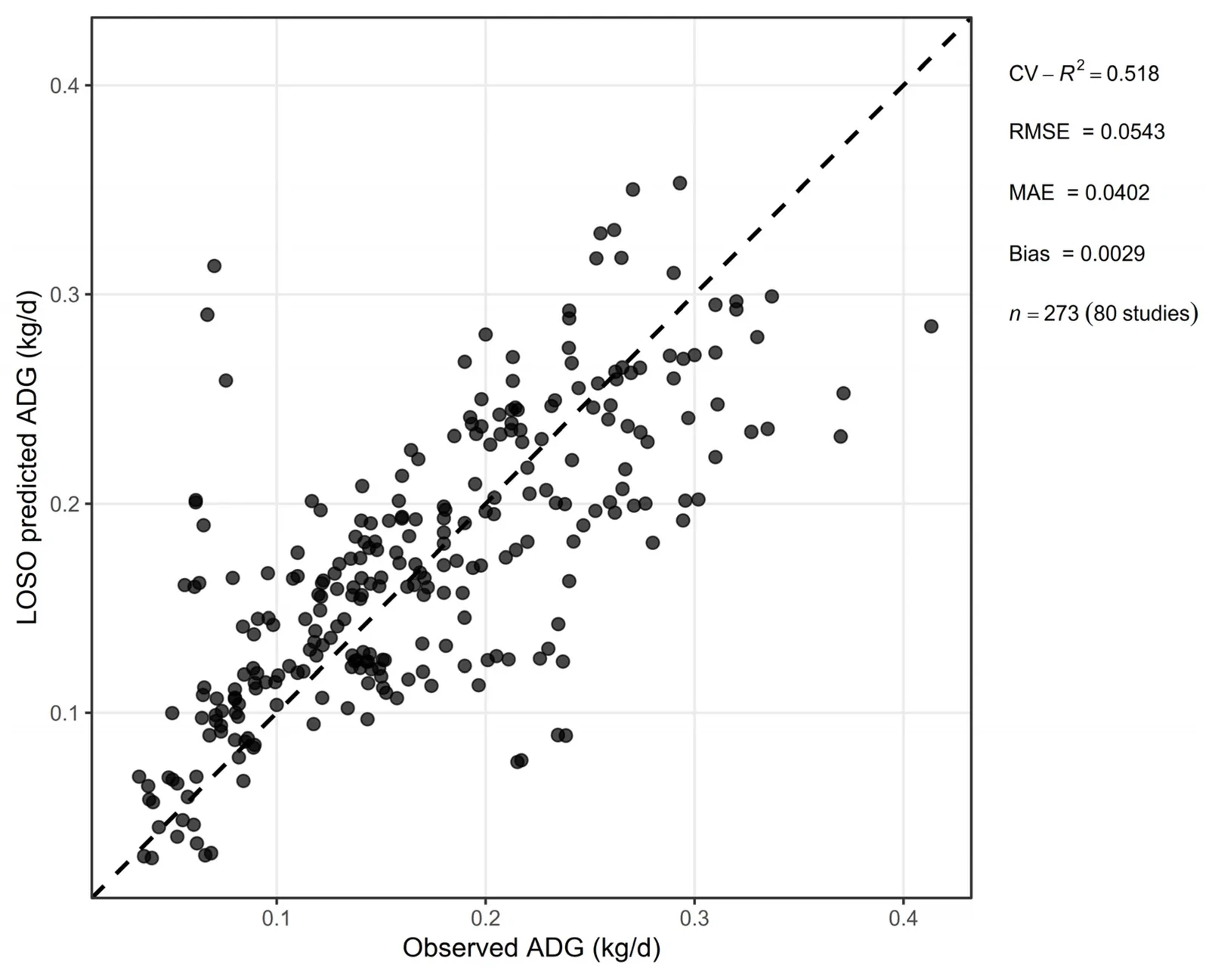
Observed vs. predicted ADG at the treatment-group level from leave-one-study-out cross-validation using the overall equation with the addition of propionic acid.

When the overall equation was used for prediction, adding propionic acid lifted CV-*R*^2^ from 0.393 to 0.518 (ΔCV-*R*^2^ = 0.124; 95% CI: 0.012–0.326; excludes zero) and cut RMSE by 10.84%, so both metrics point to clear incremental predictive value. Total VFA (ΔCV-R^2^ = 0.070; 95% CI −0.051 to 0.236) improved the point estimate, but its 95% CI crossed zero, so the improvement was not statistically significant; A:P (ΔCV-R^2^ = 0.073; 95% CI 0.003 to 0.178) improved the point estimate with an interval that barely excluded zero (Figure 4). For sheep, propionic acid raised CV-*R*^2^ to 0.485 (ΔCV-*R*^2^ = 0.056; 95% CI crosses zero) and lowered RMSE by 5.03%, an improvement whose incremental benefit is still uncertain. For goats, the same addition moved CV-*R*^2^ from −0.599 to −0.157 (ΔCV-*R*^2^ = 0.441; 95% CI: 0.035–1.337; excludes zero) and reduced RMSE by 14.92%, a relative improvement that, together with the lower RMSE, we interpret cautiously because the absolute CV-*R*^2^ remained negative, meaning the propionate-extended model still predicted goat ADG worse than the overall mean. Within both species equations, point estimates for each indicator were best when propionic acid was included (ΔCV-*R*^2^ of 0.046 and 0.204, respectively), yet both 95% CIs crossed zero; these results should be treated as exploratory. These results are presented in Table 5. Figure 5 plots observed against predicted values after propionic acid was added to the overall equation.

### 3.8. Sensitivity Analysis Results

Comparing the complete-data and full-data results, the direction of effects was identical across all four indicators, and the qualitative significance conclusions were also consistent. The Monte Carlo perturbation test (500 replicates) and the multi-inference robustness check support the main conclusion; the propionic acid interaction and the predictive increment remained directionally stable under SE perturbations. When only the two inference variants—the study-level random-effects model and the 95th-percentile precision ceiling—were considered, the directions of acetic acid, propionic acid, and total VFA were consistent with the primary analysis, but acetic acid and propionic acid no longer reached nominal statistical significance under these two variants (Table S1); this tempers the strong “robustly associated” wording used elsewhere. Funnel plots for the four VFA indicators are provided in Figures S4–S7.

### 3.9. Secondary Outcome: Feed Conversion Ratio

A parallel analysis used the feed conversion ratio (FCR, feed intake-to-gain ratio; lower values indicate better feed efficiency) as a secondary outcome, following the same two frameworks (Table S2).The unweighted LMM reproduced these directions (marginal *R*^2^ = 0.23–0.29; Table S2). In contrast to ADG, leave-one-study-out cross-validation for FCR showed only uncertain incremental prediction once VFA indicators were added (Figure S24 and Table S3; overall best ΔCV-*R*^2^ = 0.31, 95% CI −0.02 to 0.75, with the absolute CV-*R*^2^ remaining negative), and the FCR analysis yielded two significant species interactions (Figures S25 and S26, Table S4; propionate at the within-study level, *p* = 0.017; A:P at the between-study level, *p* = 0.036). Under inverse-variance weighting, higher ruminal acetic acid (*β* = −0.040, cluster-robust *p* = 0.008), propionic acid (*β* = −0.091, robust *p* < 0.001), and total VFA (*β* = −0.032, robust *p* = 0.002) were associated with a lower FCR at the within-study level, whereas the A:P ratio was positively associated with FCR at the between-study level (*β* = 0.516, robust *p* = 0.034; Table S5). The FCR results therefore agree in direction with the ADG associations but should be regarded as provisional.

## 4. Discussion

We combined 82 studies and 283 treatment groups under two complementary frameworks—inverse-variance-weighted meta-regression and multilevel mixed-effects models—to ask how total VFA, acetic acid, propionic acid, and the A:P ratio relate to average daily gain (ADG) in growing sheep and goats, and whether the VFA profile adds predictive value on top of conventional covariates. The findings separate association from prediction rather cleanly. On the association side, acetic acid, propionic acid, and total VFA were positively related to ADG in both frameworks, whereas A:P was inconsistent. On the prediction side, only propionic acid produced a clear incremental cross-study gain (ΔCV-*R*^2^ = 0.124; RMSE reduced by 10.84%), and the species-specific point estimates never translated into significant interaction effects. The rest of the discussion develops these results at three levels—mechanism, species, and application.

### 4.1. Principal Analysis Results and Mechanisms

#### 4.1.1. Separation of Correlation Strength and Predictive Increment

Across both statistical frameworks, total VFA, acetic acid, and propionic acid were significantly and positively correlated with ADG, and after adjustment for DMI, IBW, CP, NDF, and species, the direction of the effects stayed consistent with similar magnitudes (Table 3 and Table 4). This cross-method agreement supports the reproducibility of the direction of the effects. For the three indicators, the full model explained 0.625, 0.583, and 0.621 of the between-study heterogeneity, respectively. Turning to incrementality, the VFA profile added only modest heterogeneity beyond conventional covariates, though all values were positive, 0.017 for acetic acid, 0.071 for A:P, 0.107 for propionic acid, and 0.083 for total VFA, with propionic acid again the largest. LOSO reinforced this picture, showing the clearest cross-study predictive gain for propionic acid (ΔCV-*R*^2^ = 0.124; 95% CI: 0.012–0.326; RMSE reduced by 10.84%). In short, propionic acid outperformed the other variables on robust association, incremental *R*^2^, and cross-study predictive performance in leave-one-study-out validation. The concentration-based indicators (acetic acid, total VFA), by contrast, tracked the scale–mass dimension that covaries with feed intake, body size, and diet composition, so their incremental explanatory power was limited. Predictive value should therefore be judged by incremental *R*^2^ or external validation rather than by single-variable correlations, consistent with earlier studies that found VFAs relate to growth indicators yet add little predictive gain. For A:P, evidence strength differed between the two frameworks and the point estimates were small, making the result unstable. The functional form also diverged between frameworks (quadratic in Table 3; linear in Table 4), so a linear model is the most straightforward and appropriate choice in both cases. Its interpretation and application should be confined to the range of the observed data. Finally, acetic acid, propionic acid, and total VFA were analysed as absolute concentrations in mmol/L, so the interpretation of propionic acid as a relatively dilution-insensitive indicator is inferential and would benefit from confirmation by an explicit molar-proportion re-analysis.

#### 4.1.2. Mechanistic Reasoning Behind the Relationship Between Fermentation Products and Tissue Growth

For the statistical structure described above to carry meaning, every link in the chain must rest on established biochemical and physiological evidence rather than on mere correlation with phenotypes. The framework therefore runs through four interconnected processes: microbial production, epithelial absorption, portal vein and hepatic metabolism, and endocrine and tissue synthesis. Production comes first: fermentation kinetics set the metabolic profile, with each sugar molecule degraded into acetic, propionic, and butyric acids in characteristic proportions, and the propionic acid pathway competing with methanogenesis for hydrogen [3,95]. Starch-based diets favor taxa such as Succinivibrionaceae and *Prevotella*, which raises propionic acid levels [96]; elevated propionic acid is at once an ecological outcome of intense fermentation and a key precursor for hepatic gluconeogenesis. Absorption comes next. VFAs cross the epithelium by passive diffusion or through transporters such as MCT1/MCT4, NHE, and NBCe1; most butyric acid is oxidized in the epithelium to *β*-hydroxybutyrate, whereas propionic and acetic acids reach the portal vein in relatively higher proportions [97,98]. Epithelial morphology and transporter expression adapt to fermentation load as well [99,100]. One point deserves emphasis: rumen fluid concentration is not the same as substrate flux reaching the liver. Absolute concentration depends heavily on rumen fluid volume, saliva secretion, and sampling time, while the molar ratio is largely insensitive to dilution—an anatomical reason why propionic acid prediction is more robust and why concentration-based indicators are more exposed to measurement noise [101]. Metabolically, propionic acid is converted by propionyl-CoA carboxylase (PCC) to methylmalonyl-CoA and then isomerized to succinyl-CoA by methylmalonyl-CoA mutase (MCM) with vitamin B12 as a coenzyme; succinyl-CoA is subsequently converted to oxaloacetate through the forward reactions of the tricarboxylic acid cycle (succinate, fumarate, malate, and oxaloacetate), rather than by direct catalysis via PCK1 (phosphoenolpyruvate carboxykinase; also referred to as PEPCK), moved into the cytosol by the mitochondrial malate/aspartate shuttle, and finally converted by phosphoenolpyruvate carboxykinase (PCK1) to phosphoenolpyruvate (PEP), which enters gluconeogenesis to form glucose and supplies 60–74% of the carbon skeleton [5]. The same pathway upregulates hepatic gluconeogenic enzymes and has insulinotropic effects [102,103]. Chen et al. (2026) showed in a lamb oral-inoculation experiment that higher ruminal propionic acid upregulates hepatic PC and G6PC and boosts hepatic glucose output [96]; the glucose–insulin–IGF-1 axis then drives nitrogen deposition and protein synthesis. Because higher propionic acid is accompanied by a greater supply of gluconeogenic precursors, this mechanism is consistent with—but cannot by itself establish—the positive associations we observed. Feedback closes the loop: propionic acid oxidation also acts as a satiety signal (hepatic oxidation theory), so the ADG effect of propionic acid operates within a homeostatic loop of energy supply and appetite regulation, with the marginal effect flattening as concentration rises rather than growing without bound [104]; very high fermentation loads may even trigger subacute ruminal acidosis that lowers feed intake and growth [105,106]. Table 3 shows no significant quadratic term for propionic acid, so within the observed range the relationship looks predominantly linear. Still, the mechanisms above hint that endogenous effects of very high propionic acid may decelerate. This logic explains both the strong concentration-based correlations we observed and the prediction of stable structural configurations, the stability being owed to the measurement configuration rather than to the strength of the biological associations.

### 4.2. Species Differences and Cross-Study Predictive Value

The species-specific slopes follow a point-estimation pattern in which acetic acid is the more sensitive indicator for sheep and propionic acid for goats, yet none of the Species × X interaction terms reached statistical significance. We therefore treat these results as exploratory rather than fitting separate models per species. Variation in microbial community structure among sheep breeds may also weaken the stability of the species-specific signal [107]. The pattern fits with the host’s dual control over fermentation dynamics: sheep and goats differ in their feeding and rumination behaviour [108], and goats have a relatively larger rumen volume and more flexible retention times, so the effective fermentation profile may differ between species even on identical diets. The host genome is a major determinant of the core microbiota and markedly shapes the rumen microbial community [4,109], and the microbial response to dietary change is fairly host-specific [110,111]. The non-significant interaction is consistent with Salah et al. (2015), who reported that intake responses in sheep, goats, and cattle run parallel but differ in intercepts, with systematic between-species differences appearing as baseline rather than slope differences [112]. A unified framework with species-level effects is therefore preferable to separate models per species. On a point-estimation basis, LOSO indicated that propionic acid was the optimal additive component in every predictive scenario: the gain in overall prediction was clear (ΔCV-*R*^2^ = 0.124; 95% CI excludes zero; RMSE reduced by 10.84%), and the improvement was largest for goats (ΔCV-*R*^2^ = 0.441; RMSE reduced by 14.92%) despite a weak baseline; in all other scenarios, the 95% CI crossed zero. It is worth noting that the incremental gain was greatest for targets with weaker baselines: when conventional covariates fail to capture the underlying pattern, configuration indicators may carry more marginal value, and such an inverse relationship between predicted gain and baseline performance is visible only through leave-one-study-out cross-validation.

### 4.3. Comparison with Previous Studies and Robustness

A number of independent findings support the positive correlation we observed between propionic acid and ADG. Lambs given calcium propionate grew better in a meta-analysis [7]; goats with high daily weight gain carried higher ruminal propionic acid levels [8]; high-yielding Hu sheep had elevated ruminal propionate [9]; yeast-based supplements that raised ruminal propionate and total VFA improved ADG in goats [10]; post-weaning ADG in lambs was tied to ruminal microbiota composition [11]; and an oral administration experiment confirmed that increased ruminal propionate upregulates key hepatic gluconeogenic enzymes [96]. The reverse pattern was also observed: Velázquez-Cruz et al. (2024) found that low doses (10 g/kg DM) of calcium or sodium propionate failed to improve growth in fattening lambs [113], matching our view that the effects depend on dose and physiological stage—the growth-promoting action of propionate is not unconditional, and the ADG response to propionic acid should be read as the net result of the fermentation–metabolism chain rather than of the additive alone. Earlier studies generally reported effects through correlation or regression *p*-values, while few provided incremental *R*^2^, robust inference, and leave-one-out validation together. The present study addresses that gap by reporting the limited explanation of incremental heterogeneity transparently and by using LOSO to quantify the cross-study predictive benefit of propionic acid directly. Robustness checks support the same conclusions: the inverse-variance framework (Table 3) and the mixed linear model framework (Table 4) agreed on the significant positive correlation of acetic acid, propionic acid, and total VFA, and when the A:P direction was treated as non-robust, neither the complete-versus-full dataset comparison, nor the SE Monte Carlo simulation (500 replicates), nor the multi-simulation inference changed the direction or qualitative conclusion (Result 3.8), indicating that the primary findings do not hinge on specific data-cleaning or statistical decisions.

### 4.4. Application Significance and Limitations

The concentration- and composition-based approaches described above—designed to capture fermentation intensity and compositional stability in prediction—point to a differentiated application strategy. Total VFA and acetic acid work as monitoring indicators of fermentation intensity, suited to tracking fermentation scale in ration formulation and livestock management. Propionic acid emerges as the more robust compositional indicator across studies (overall equation prediction ΔCV-*R*^2^ = 0.124, RMSE reduced by 10.84%), which agrees with evidence that propionic acid production improves feed efficiency and growth performance [9,10,11,96]. A:P, in turn, should not be used alone to predict growth performance. The total–propionic acid model can serve as an empirical estimation model built on treatment-group means and checked through leave-one-out internal validation; it estimates mean performance across treatment groups under similar experimental conditions, but it does not support precise individual prediction and calls for independent external validation. Some design limitations should be kept in mind when interpreting these results. Because the findings rest on treatment-group means, publication bias is a real risk; funnel plots and Egger’s regression flagged publication bias for all four indicators (Egger’s *p*-values < 0.001; see Table S1), which may have inflated the association strength. Heterogeneity arising from breed, feeding practices, sampling times, and VFA determination methods was not fully accounted for [101], and the total VFA dataset covered only 279 treatment groups across 81 studies, with the functional-form choice sensitive to the analytic framework. Leave-one-out validation is internal validation, so the model’s predictive power for baseline values in goats is relatively weak and does not justify developing or relying on goat-specific equations. Taken together, these limitations argue that the quantitative inferences should be read at the treatment-group (aggregate) level, not at the individual-animal level, and with caution when extrapolating to individual animals.

## 5. Conclusions

This study used a multilevel modeling and cross-validation framework to assess how well the VFA profile predicts ADG. Total VFA, acetic acid, and propionic acid were robustly associated with ADG in both analytical frameworks; beyond conventional covariate adjustment, however, the VFA profile added only limited incremental explanatory power for inter-study heterogeneity. Among the components, propionic acid showed the highest incremental explanatory value and the most stable additional contribution at the cross-study prediction level, whereas A:P was neither robust nor independently informative.

Point estimates differed between sheep and goats, but the differences were not statistically significant at the interaction level. We therefore recommend incorporating propionic acid into a growth prediction model based on treatment-group means, with priority given to validation using independent external data; application of such models should be restricted to estimates derived from treatment-group means under conditionally similar settings. Future research should combine mechanistic validation with a richer set of host covariates to deepen our understanding of VFA-mediated growth regulation.

## Figures and Tables

**Table 1:** Data and references of the 82 studies in the complete-case analysis.

Species	Reference	Treatment	Basic Information of Sheep and Goats
Sheep/Goat	Xueyan Lin et al., 2023 [4]	Feeding diets with different concentrate-to-roughage ratios to both sheep and goats.	Male Small-tailed Han sheep and male Boer goats aged 5 months (33.87 ± 1.70 kg).
sheep	Fu Jian et al., 2024 [14]	Natural grazing versus combined grazing with supplemental feeding.	Grassland Tibetan sheep aged 7 months, with a body weight of (23.74 ± 2.16) kg.
sheep	Guo Yaya, 2025 [15]	Feeding ad libitarily a total mixed ration with equal energy and nitrogen content, formulated with leaves of blackberry wolfberry at varying concentrations.	Du-hu crossbred lambs, aged 3 months, weighing 29.58 ± 2.06 kg.
sheep	Zhang Minghui, 2025 [16]	Feeding whole mixed particle diets with varying levels of *Scutellaria baicalensis* straw under conditions of equal energy and nitrogen content.	Male Bayinbuluk sheep, aged 4 to 5 months, weighing 27.08 ± 3.12 kg.
sheep	Kang Yong, 2025 [17]	Feeding a complete mixed ration supplemented with alfalfa hay or alfalfa silage as roughage.	Hulunbuir sheep aged 3–5 months.
sheep	Chen Yanxu et al., 2023 [18]	Feeding a total mixed ration with equal energy and nitrogen content supplemented with chrysanthemum meal at varying levels.	Hybrid male lambs with a body weight of (28.30 ± 3.95) kg.
sheep	Xie Biao et al., 2017 [19]	Feeding livestock with feed supplemented with different levels of jujube powder.	Hybrid male lambs.
sheep	Wei Dalian et al., 2024 [20]	Adding fermented Agrocybe aegerita mushroom meal at varying levels to the basal diet.	Dantui Lake Sheep (equal numbers of males and females).
sheep	Wang Xingxing, 2025 [21]	Add a 4% brown algae extract to the diet.	Hybrid male lambs aged 3 months.
sheep	Qi Shuai et al., 2025 [22]	Replacing the original soybean straw in the diet with different concentrations of fermented soybean straw.	Male Hu lamb aged 4 months, weighing (27.00 ± 2.00) kg.
sheep	Zhang Weihua, 2025 [23]	High-nutrient diets with different energy and protein levels.	Male Hu lambs with a body weight of (20.89 ± 0.54) kg.
sheep	Jian Zhao et al., 2024 [24]	Feeding two total mixed rations.	Male Tibetan sheep aged 5 months, with a body weight of (29.37 ± 1.16) kg.
sheep	Yao Wang et al., 2022 [25]	With or without dietary supplementation of *Pichia kudriavzevii*.	Hu sheep aged (108 ± 5) days, with a body weight of (28.32 ± 0.71) kg.
sheep	Liang Chen et al., 2024 [26]	Five dietary treatments.	Native breed male lambs aged 3 months, with a body weight of (28.24 ± 1.80) kg.
sheep	Wang Lele et al., 2023 [27]	Basal diet supplemented with different levels of fermented wood chip fungi.	Male Altay sheep aged 4 months.
sheep	Maria M. Della Rosa et al., 2022 [28]	Feeding a ryegrass-based forage diet supplemented with different levels of forage rape.	Lambs with a body weight of (42 ± 0.4) kg.
sheep	Jin Yaxing, 2024 [29]	Feeding total mixed rations with different concentrate-to-roughage ratios.	Male Duolang sheep aged 3–4 months, with a body weight of (24.06 ± 3.10) kg.
sheep	Wei Deshuang, 2025 [30]	Three diets with different dry matter contents.	Healthy male Hu sheep aged 4 months.
sheep	Chen Binglong, 2025 [31]	Different combinations and addition ratios of microbial strains and enzymes.	Male Bashbay lambs aged 4 months, with an average body weight of approximately 29 kg.
sheep	Jiang Yaxin, 2023 [32]	Different proportions of pine needles in the fermentation substrate.	Male Kazakh sheep with a body weight of (30.30 ± 0.36) kg.
sheep	Zhang Duihong, 2021 [33]	Feeding different levels of *Aspergillus oryzae* culture.	Hu sheep aged 3 months, with a body weight of (20.18 ± 1.89) kg.
sheep	Yuyang Xue et al., 2024 [34]	Different levels of non-starch polysaccharide enzymes.	Female fattening crossbred sheep aged 5 months.
sheep	Zhu Aoxiang et al., 2017 [35]	Feeding total mixed rations with different selenium sources and selenium levels.	Female Hu sheep aged 6 months, with an average body weight of (27.95 ± 0.27) kg.
sheep	Abiodun Mayowa Akanmu et al., 2026 [36]	Four different dietary treatments.	Male Merino lambs aged 4 months, with a body weight of (28.8 ± 0.4) kg.
sheep	Jianxin Jiao et al., 2025 [37]	Feeding three different corn varieties.	Female Hu sheep aged 4 months, with a body weight of (19.6 ± 0.26) kg.
sheep	Xiaolin Wang et al., 2024 [38]	Feeding diets with different metabolizable energy contents.	Yunnan semi-fine wool sheep aged 10 months, with a body weight of (30.8 ± 1.9) kg.
sheep	Xiaowen Ma et al., 2022 [39]	Four experimental diets with different starch sources.	Hu lambs with a body weight of (29.70 ± 1.70) kg.
sheep	Guangyuan Tian et al., 2026 [40]	Basal diet supplemented with different levels of phytosterols on a dry matter basis.	Male fattening lambs aged 4 months, with a body weight of (23.90 ± 0.83) kg.
sheep	Zhang Yanzhen et al., 2024 [41]	Different residual feed intake.	Hu sheep aged (97 ± 6.1) days, with a body weight of (23.33 ± 3.58) kg.
sheep	Huwei Zhao et al., 2025 [42]	Supplementation with different levels of rubber seed cake.	Hu sheep aged 3 months, with a body weight of 17.01 ± 0.57 kg.
sheep	M. Mahmoudi-Abyane et al., 2020 [43]	Diet supplemented with different nitrogen sources.	Male lambs with a body weight of (34.7 ± 1.8) kg.
sheep	Shiqi Zhang et al., 2025 [44]	Feeding a basal diet supplemented with different levels of guanidinoacetic acid.	Male Kazakh sheep aged 5 months, with a body weight of (34.29 ± 1.95) kg.
sheep	Xinlong Zhang et al., 2026 [45]	Replacing soybean meal with different levels of vitamin B.	Male Hu lambs aged (80 ± 4) days, with a body weight of (26.5 ± 0.59) kg.
sheep	Uriel Hidalgo-Hernández et al., 2025 [46]	Feeding different doses of glycerol.	Crossbred lambs aged 60 days, with a body weight of (25 ± 5) kg.
sheep	GUO Yun-xia et al., 2023 [47]	Replacing dietary components with different concentrations of yam concentrate.	Male Small-tailed Han lambs aged 60 days, with a body weight of (22.68 ± 2.56) kg.
sheep	T. Ran et al., 2021 [48]	Different conditioning temperatures of pelleted total mixed ration.	Lambs aged (120 ± 10.2) days, with a body weight of (24.9 ± 3.3) kg.
sheep	Cristina Saro et al., 2020 [49]	Feeding diets with different crude protein contents.	Male Assaf lambs aged (89 ± 0.8) days, with a body weight of (30 ± 1.9) kg.
sheep	Ma Dongmei, 2023 [50]	Whether the diet was supplemented with Jerusalem artichoke polysaccharide.	Weaned sheep aged 3 months, with a body weight of (26 ± 2) kg.
sheep	Niu Shenghua et al., 2026 [51]	High-concentrate diet supplemented with 2.2% Chao San Xian.	Fattening sheep aged 3 months, with an average body weight of (21.66 ± 1.88) kg.
sheep	Gao Xiujie et al., 2025 [52]	Basal diet supplemented with different levels of *Macleaya cordata* extract.	Fattening male Hu lambs with an average body weight of (27.56 ± 0.24) kg.
sheep	Henan Lu et al., 2025 [53]	Replacing different proportions of soybean meal with urea, cottonseed meal, and rapeseed meal under isoenergetic and isonitrogenous conditions.	Male Dumeng lambs aged 3 months, with an average body weight of (23.29 ± 0.87) kg.
sheep	Borui Han et al., 2026 [54]	Basal diet supplemented with 0.3 g/d active dry yeast.	Duhan lambs aged 4 months, with an average body weight of (29.03 ± 0.45) kg.
sheep	Hatem A. Hamdon et al., 2026 [55]	Diet supplemented with different levels of *Saccharomyces cerevisiae*.	Farafra lambs aged 5–6 months (under desert conditions), with an average body weight of (26.7 ± 1.83) kg.
sheep	Mehri Montazerharzand et al., 2025 [56]	Diet supplemented with 1% walnut shell biochar.	Male Qezel lambs aged 2–3 months, with an average body weight of (34.5) kg.
sheep	Xie Yining et al., 2026 [57]	Comparison of two rearing systems: grazing versus confinement feeding.	Male Gangba sheep aged 4 months, with an average body weight of (16.28) kg.
sheep	Wang Chuying et al., 2025 [58]	Diet supplemented with 30 g/d yeast culture under feed restriction.	Suffolk sheep aged 4 months, with an average body weight of (22 ± 0.5) kg.
goat	Wang Boyao, 2023 [59]	Three experimental diets formulated with different types of soybean meal.	Male Liaoning cashmere goat lambs aged 3 months, with a body weight of (11.28 ± 0.7) kg.
goat	Su Hongping et al., 2025 [60]	Basal diet supplemented with different levels of nano-selenium.	Male Shanbei white cashmere goat lambs aged 5 months, with an average body weight of (22.0 ± 1.5) kg.
goat	Bao Siqingaowa, 2018 [61]	Diet supplemented with 3% *Artemisia ordosica*.	Weaned castrated male lambs aged 4 months.
goat	Zhang Lu, 2023 [62]	Diet supplemented with tannins from different sources.	Female Liaoning cashmere goats aged 18 months, with a body weight of (32.59 ± 2.15) kg.
goat	Luo Dike, 2025 [63]	Replacing different levels of the diet with fermented green tea residue.	Male Shanbei white cashmere goats aged 6 months, with an average body weight of (23.89 ± 1.65) kg.
goat	Yu Hao, 2024 [64]	With or without dietary supplementation of noni fruit flavonoids.	Healthy male Albas white cashmere goats aged 4 months.
goat	Hu Zhichao, 2020 [65]	Diet supplemented with different levels of silaged paper mulberry and cottonseed meal.	Healthy female meat-purpose goats aged 4 months, weighing approximately 23 kg.
goat	Huang Wenqin et al., 2018 [66]	Replacing different proportions of whole-plant corn silage with whole-plant sugarcane.	Weaned goats aged 4–5 months.
goat	Zhao Lichao, 2023 [67]	Supplementation with different artificial sweeteners.	Young dairy goats with an average body weight of (34.90 ± 0.68) kg.
goat	Qu Bo et al., 2020 [68]	With or without dietary rumen leucine supplementation.	Healthy young Guanzhong dairy goats.
goat	Tan Zehao, 2025 [69]	Whether the diet was supplemented with bee pollen.	Growing Jintang black goats with an average body weight of (33.36 ± 1.34) kg.
goat	Zhao Xingrui et al., 2025 [70]	Diet supplemented with creatine pyruvate and coated γ-aminobutyric acid.	Nubian black goats aged 4 months, with an average body weight of (22.61 ± 0.47) kg.
goat	Xu Qian et al., 2024 [71]	Diet supplemented with different levels of garlic skin.	Male castrated Fuqing goats aged 9 months, with an average body weight of (15.42 ± 1.04) kg.
goat	Yang Yuntian, 2021 [72]	Basal diet supplemented with different levels of *Saccharomyces cerevisiae*.	Female Guanzhong dairy goats aged 4 months, with a body weight of (19.65 ± 0.41) kg.
goat	Xinhong Zhou et al., 2025 [73]	Whether the diet was supplemented with cecropin.	Male Yudong black goats aged 3 months, with a body weight of (23.19 ± 0.32) kg.
goat	Han Yong et al., 2008 [74]	Diets supplemented with POBF, probiotics, or POBF + probiotics, respectively.	Growing crossbred goats aged 5 months, with a body weight of (13.4 ± 1.3) kg.
goat	Liyuan Cai et al., 2023 [75]	Diet supplemented with different proportional combinations of *Saccharomyces cerevisiae* and *Clostridium butyricum*.	Crossbred goats aged (5.0 ± 1.0) months, with a body weight of (19–21) kg.
goat	Ngo Thi Minh Suong et al., 2022 [76]	Whether to feed a total mixed ration containing 50% anthocyanin-rich black sugarcane.	Male Thai native × Nubian crossbred goats, weighing (14.42 ± 0.6) kg.
goat	Nittaya Taethaisong et al., 2023 [77]	Feeding concentrate feeds containing different levels of neem leaves and polyethylene glycol.	Male Anglo-Nubian goats with a body weight of (20 ± 2.0) kg.
goat	Jia Haobin et al., 2026 [78]	Feeding diets with different concentrate-to-roughage ratios.	Guangfeng goats aged (120 ± 7) days.
goat	Pan Li, 2022 [79]	The basal diet was supplemented with 2% whole Jerusalem artichoke powder.	Crossbred Boer male goats aged 3 months, with a body weight of (26 ± 3) kg.
goat	Chunhui Wang et al., 2026 [80]	Diets formulated with varying crude protein contents.	Chuannan black goats aged 4 months, with a body weight of (13.75 ± 0.27) kg.
goat	Lingling Xie et al., 2025 [81]	Supplementation with varying levels of Hu-song extract.	Guizhou black goats with a body weight of (16.03 ± 0.79) kg.
goat	Saeid M. Basmaeil et al., 2023 [82]	Diet supplemented with different concentrations of lasalocid.	Male Ardi goats aged 3 months, with an average body weight of (17.12 ± 0.51) kg.
goat	Yong Long et al., 2024 [83]	Whether to add mushroom residue.	Guizhou black goats with an average initial body weight of (22.41 ± 0.90) kg.
goat	Liyuan Cai et al., 2021 [84]	Whether to supplement with *Clostridium butyricum*.	Female crossbred goats aged (6 ± 1) months, with a body weight of (23.23 ± 3.10) kg.
goat	Ligang Xue et al., 2022 [85]	Basal diet supplemented with different levels of *Clostridium butyricum* and *Saccharomyces cerevisiae*.	Male Macheng black goats, weighing (22.25 ± 4.26) kg.
goat	Ali Mujtaba Shah et al., 2019 [86]	Diet supplemented with different levels of tea plant.	Nanjiang yellow goats aged 6 months, with a body weight of (34.6 ± 4.16) kg.
goat	Kang Yang et al., 2021 [87]	Feeding diets with different levels of dietary cation–anion difference.	Female Qianbei multi-colored goats aged 13 months, with a body weight of (30.07 ± 0.55) kg.
goat	Ashvini Pundalik Bansod et al., 2026 [88]	Diet supplemented with different levels of cauliflower leaf powder.	Unspecified breed male goats aged 6–8 months, with an average initial body weight of (10.96 ± 2.21) kg.
goat	A. Zali et al., 2019 [89]	Diet supplemented with different levels of distiller’s grains.	Male goats aged 5–6 months, with a body weight of (20 ± 2) kg.
goat	Gong Zhengfa et al., 2026 [90]	Feeding a basal diet supplemented with allicin.	Qianbei Ma goats with a body weight of (22.80 ± 1.06) kg.
goat	Cai Liyuan et al., 2026 [91]	Feeding a basal diet supplemented with a co-culture of *Saccharomyces cerevisiae* and *Rhodotorula glutinis.*	Goats aged (90 ± 3) days, with a body weight of (10.21 ± 1.10) kg.
goat	Chen Yan et al., 2025 [92]	Diet supplemented with different proportions of potato starch residue–corn mixed fermented feed.	Female Boer goats aged 3 months, with a body weight of (25.17 ± 1.34) kg.
goat	Yuan Ruitong, 2025 [93]	Diet supplemented with different levels of chestnut tannin and tannic acid.	Female Liaoning cashmere goats aged 6 months, with a body weight of (26.34 ± 4.69) kg.
goat	Caixia Zhang et al., 2025 [94]	Diet supplemented with non-protected or rumen-protected *Clostridium butyricum*.	Fattening Albas goats aged 7–8 months, with an average body weight of (21.94 ± 2.07) kg.

**Table 2:** Variable Range.

Population	Variable	*n*	Mean	SD	Median	Min	Max
Overall	ADG	283	0.170	0.081	0.159	0.034	0.413
	acetic acid	290	48.297	22.696	46.830	10.147	225.510
	propionic acid	290	17.951	9.829	15.785	3.160	79.420
	A:P	290	3.055	1.150	3.060	0.970	6.580
	VFA	281	77.268	36.771	74.100	15.785	368.910
	DMI	290	1.155	0.418	1.130	0.327	2.320
	IBW	290	24.788	6.549	24.900	10.213	42.000
	CP	290	14.918	5.598	14.090	6.480	60.650
	NDF	290	39.063	9.734	39.210	15.400	65.610
Sheep	ADG	157	0.213	0.073	0.207	0.061	0.413
	acetic acid	164	54.138	25.011	51.780	13.420	225.510
	propionic acid	164	20.843	10.431	18.045	6.126	79.420
	A:P	164	2.990	1.146	2.990	0.970	6.580
	VFA	155	86.318	42.025	79.910	30.990	368.910
	DMI	164	1.428	0.315	1.444	0.830	2.320
	IBW	164	27.326	4.923	27.555	16.210	42.000
	CP	164	14.445	2.511	14.075	6.480	24.400
	NDF	164	38.488	8.463	38.460	17.300	62.910
Goat	ADG	126	0.117	0.056	0.103	0.034	0.238
	acetic acid	126	40.695	16.497	41.556	10.147	85.690
	propionic acid	126	14.187	7.499	13.537	3.160	60.207
	A:P	126	3.155	1.155	3.070	1.290	6.140
	VFA	126	66.135	25.026	64.365	15.785	155.640
	DMI	126	0.830	0.265	0.860	0.327	1.535
	IBW	126	21.484	6.935	21.940	10.213	34.900
	CP	126	15.534	7.972	14.210	8.126	60.650
	NDF	126	39.812	11.165	41.720	15.400	65.610

*n* indicates the number of treatment groups with the variable reported. Total VFA was available for 281 groups in the descriptive statistics but analyses of total VFA used 279 groups (81 studies), with the difference arising from 2 sheep groups excluded because of missing covariate or standard-error data.

**Table 3:** Association analysis between predictive factors and ADG using inverse-variance multilevel meta-regression.

Metric Variable	Model Type	Studies	Group Count	*β* (95%CI)	Standardization Coefficient	Robust *p*-Value	Incremental *R*^2^	Heterogeneity *R*^2^
Overall—Acetic acid	Linear	82	283	0.00088 (0.00031, 0.00144)	0.0201	0.003	0.017	0.583
Overall—Propionic acid	Linear	82	283	0.00188 (0.00095, 0.00280)	0.0186	<0.001	0.107	0.621
Overall—A:P	Quadratic	80	277	−0.00820 (−0.01720, 0.00080)	−0.0095	0.074	0.071	0.607
Overall—Total VFA	Quadratic	81	279	0.00088 (0.00042, 0.00135)	0.0325	<0.001	0.083	0.625
Sheep—Acetic acid	Linear	46	157	0.00098 (0.00022, 0.00173)	0.0246	0.013	−0.017	0.454
Sheep—Propionic acid	Linear	46	157	0.00148 (0.00011, 0.00284)	0.0156	0.035	0.055	0.493
Sheep—A:P	Quadratic	45	154	−0.00157 (−0.01167, 0.00852)	−0.0018	0.754	0.006	0.449
Sheep—Total VFA	Quadratic	45	153	0.00108 (0.00042, 0.00175)	0.0458	0.002	0.028	0.521
Goat—Acetic acid	Linear	37	126	0.00052 (−0.00030, 0.00134)	0.0086	0.205	0.014	0.173
Goat—Propionic acid	Linear	37	126	0.00227 (0.00116, 0.00338)	0.0170	<0.001	0.100	0.245
Goat—A:P	Quadratic	36	123	−0.01575 (−0.02834, −0.00315)	−0.0182	0.016	0.154	0.315
Goat—Total VFA	Quadratic	37	126	0.00055 (0.00005, 0.00105)	0.0138	0.033	0.042	0.197

*β* represents the inverse-variance-weighted meta-regression coefficient: for acetic acid, propionic acid, and total volatile fatty acids (VFAs), the units are mmol/L; A:P denotes the unit ratio; *β* per SD indicates the change in daily gain (ADG) corresponding to an increase of 1 standard deviation in the predictor variable. The Robust *p*-value is the cluster-Robust *p*-value (CR1, with studies as the cluster unit). Incremental *R*^2^ is the additional heterogeneity explained by the complete model relative to the covariates-only model (dry matter intake (DMI)/initial body weight (IBW)/crude protein (CP)/neutral detergent fiber (NDF); the full sample also includes species), whereas heterogeneity *R*^2^ is the proportion of heterogeneity explained by the complete model relative to the null model. The number of studies refers to the total number of independent studies; the number of groups refers to the total number of treatment groups across all analyses.

**Table 4:** Complementary verification of predictors for ADG using a linear mixed-effects model (LMM).

Metric Variable	Studies	Group Count	*β* (95%CI)	*p*-Value	Marginal *R*^2^
Overall—Acetic acid	82	283	0.00070 (0.00034, 0.00106)	<0.001	0.610
Overall—Propionic acid	82	283	0.00213 (0.00139, 0.00287)	<0.001	0.637
Overall—A:P	80	277	−0.00769 (−0.01384, −0.00153)	0.015	0.615
Overall—Total VFA	81	279	0.00127 (0.00080, 0.00174)	<0.001	0.642
Sheep—Acetic acid	46	157	0.00066 (0.00022, 0.00110)	0.004	0.500
Sheep—Propionic acid	46	157	0.00157 (0.00061, 0.00254)	0.002	0.516
Sheep—A:P	45	154	−0.00528 (−0.01366, 0.00311)	0.220	0.479
Sheep—Total VFA	45	153	0.00157 (0.00092, 0.00223)	<0.001	0.556
Goat—Acetic acid	37	126	0.00063 (−0.00003, 0.00129)	0.065	0.352
Goat—Propionic acid	37	126	0.00279 (0.00157, 0.00401)	<0.001	0.391
Goat—A:P	36	123	−0.00883 (−0.01776, 0.00011)	0.056	0.377
Goat—Total VFA	37	126	0.00148 (0.00009, 0.00286)	0.039	0.354

*β* denotes the fixed-effects coefficient in the LMM (units are consistent with Table 3); *p* value represents the within-model *p* value calculated using the Satterthwaite approximation. The marginal *R*^2^ is calculated using Nakagawa and Schielzeth’s method (fixed-effects variance/total variance). The “Number of studies” refers to the total number of independent studies; the “Number of groups” refers to the total number of treatment groups across all analyses.

**Table 5:** Incremental predictive value of leave-one-study-out (LOSO) cross-validation.

Validation Scenario	Number of Groups	Baseline CV-*R*^2^	Propionic Acid-Model CV-*R*^2^	ΔCV-*R*^2^ (Propionic Acid vs. Baseline, 95% CI)	RMSE Reduction (%)	Propionic Acid *p*
Overall → Overall	273	0.393	0.518	0.124 (0.012,0.326)	10.84	<0.001
Overall → Sheep	150	0.429	0.485	0.056 (−0.056,0.165)	5.03	<0.001
Overall → Goat	123	−0.599	−0.157	0.441 (0.035,1.337)	14.92	<0.001
Sheep → Sheep	150	0.384	0.431	0.046 (−0.052,0.160)	3.82	<0.001
Goat → Goat	123	−0.250	−0.047	0.204 (−0.061,0.547)	8.51	<0.001

In the column headings, A → B denotes the model trained on dataset A and validated on dataset B; the baseline model contained DMI, IBW, CP and NDF (plus species for the total sample); LOSO provides internal validation.; ΔCV-*R*^2^ = CV-*R*^2^ (with propionic acid) − baseline CV-*R*^2^ (without propionic acid); the 95% CI was derived from study-level paired bootstrap (1000 resamples; 2.5% and 97.5% percentiles); bold indicates that the 95% CI excludes 0. N denotes the number of treatment groups predicted in each validation direction; *P* for propionic acid is the *P*-value of the propionic acid coefficient in the LMM that included propionic acid. A negative baseline CV-*R*^2^ indicates that the baseline model predicted worse than simply using the overall mean, reflecting the weak baseline predictive ability for goats.

## Data Availability

The datasets analyzed during the current study are available from the corresponding author on reasonable request. Analysis scripts and intermediate results are provided in the Supplementary Materials.

## Supplementary Materials

The following supporting information can be downloaded at https://www.mdpi.com/article/10.3390/ani16213386/s1, Supplementary Text S1: Search and screening log; Figures S1–S3: Inverse-variance multilevel meta-regression forest plots for the VFA–ADG associations (total, sheep, and goat populations); Figures S4–S7: Funnel plots for acetate, the acetate-to-propionate (A:P) ratio, propionate, and total VFA; Table S1: Publication bias and sensitivity analyses; Figures S8–S23 and Table S2: Complete results of the parallel feed conversion ratio (FCR) analysis (forest plots, linear mixed models, leave-one-study-out cross-validation, incremental CV-*R*^2^, and observed-vs-predicted plots); Figure S24 and Table S3: Leave-one-study-out cross-validation results for the FCR analysis; Figures S25 and S26 and Table S4: Species-interaction results for the FCR analysis; Table S5: Within/between sensitivity analyses for the FCR analysis; Table S6: Bibliography of the included studies; Table S7: Adapted risk-of-bias checklist. PRISMA 2020 checklist: completed checklist for this review. Table S8: Covariate Diagnostics (Variance Inflation Factors and Model Comparisons).

## Abbreviations

VFA, volatile fatty acid; A:P, the acetate-to-propionate ratio; ADG, average daily gain; DMI, dry matter intake; IBW, initial body weight; CP, crude protein; NDF, neutral detergent fiber; LMM, linear mixed model; LOSO, leave-one-study-out; CV-R^2^, cross-validated coefficient of determination; RMSE, root-mean-square error; CR1, cluster-robust standard errors; BH, Benjamini–Hochberg; AIC, Akaike information criterion; VIF, variance inflation factor; FCR, feed conversion ratio; Q-test, Cochran’s Q test; τ^2^, between-study variance.
