# Supplementary material for: Association of Ruminal Volatile Fatty Acids with Average Daily Gain in Growing Sheep and Goats: A Systematic Review and Meta-Regression Analysis
Source: Animals (Basel). 2026 Oct 27;16(21):3386. doi: 10.3390/ani16213386 (PMC13761521; doi:10.3390/ani16213386)
Supplement: Supplementary file 1 [file animals-16-03386-s001.zip › S1 Methods and Forest Plots/Figure S1 ADG forest plot (total).pdf]

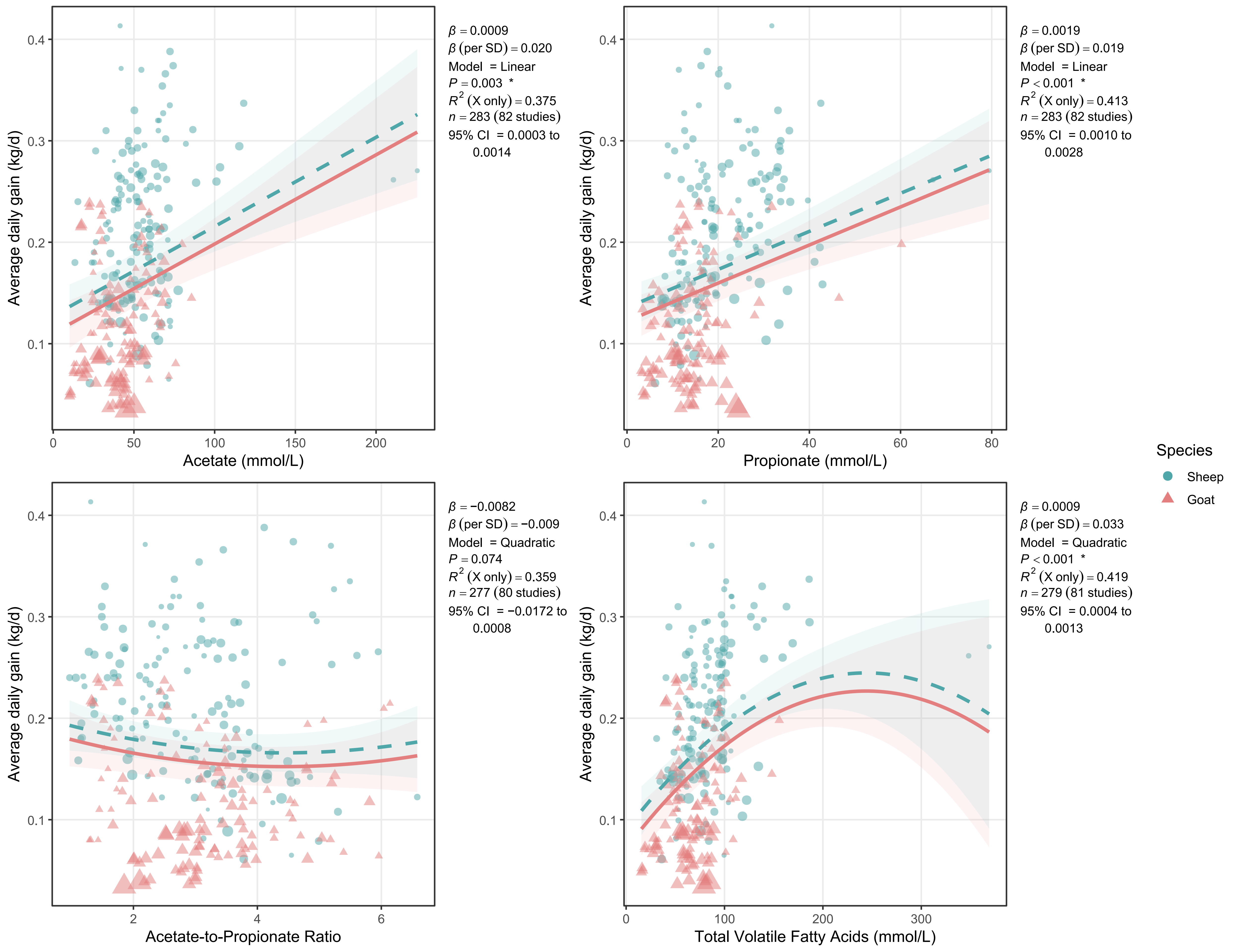

β = 0.0009
β (per SD) = 0.020
Model = Linear
P = 0.003 *
R2 (X only) = 0.375
n = 283 (82 studies)
95% CI = 0.0003 to 0.0014
Average daily gain (kg/d)
Acetate (mmol/L)
β = 0.0019
β (per SD) = 0.019
Model = Linear
P < 0.001 *
R2 (X only) = 0.413
n = 283 (82 studies)
95% CI = 0.0010 to 0.0028
Average daily gain (kg/d)
Propionate (mmol/L)
Species
Sheep
Goat
β = −0.0082
β (per SD) = −0.009
Model = Quadratic
P = 0.074
R2 (X only) = 0.359
n = 277 (80 studies)
95% CI = −0.0172 to 0.0008
Average daily gain (kg/d)
Acetate-to-Propionate Ratio
β = 0.0009
β (per SD) = 0.033
Model = Quadratic
P < 0.001 *
R2 (X only) = 0.419
n = 279 (81 studies)
95% CI = 0.0004 to 0.0013
Average daily gain (kg/d)
Total Volatile Fatty Acids (mmol/L)
