# Supplementary material for: Association of Ruminal Volatile Fatty Acids with Average Daily Gain in Growing Sheep and Goats: A Systematic Review and Meta-Regression Analysis
Source: Animals (Basel). 2026 Oct 27;16(21):3386. doi: 10.3390/ani16213386 (PMC13761521; doi:10.3390/ani16213386)
Supplement: Supplementary file 1 [file animals-16-03386-s001.zip › S1 Methods and Forest Plots/Figure S2 ADG forest plot (sheep).pdf]

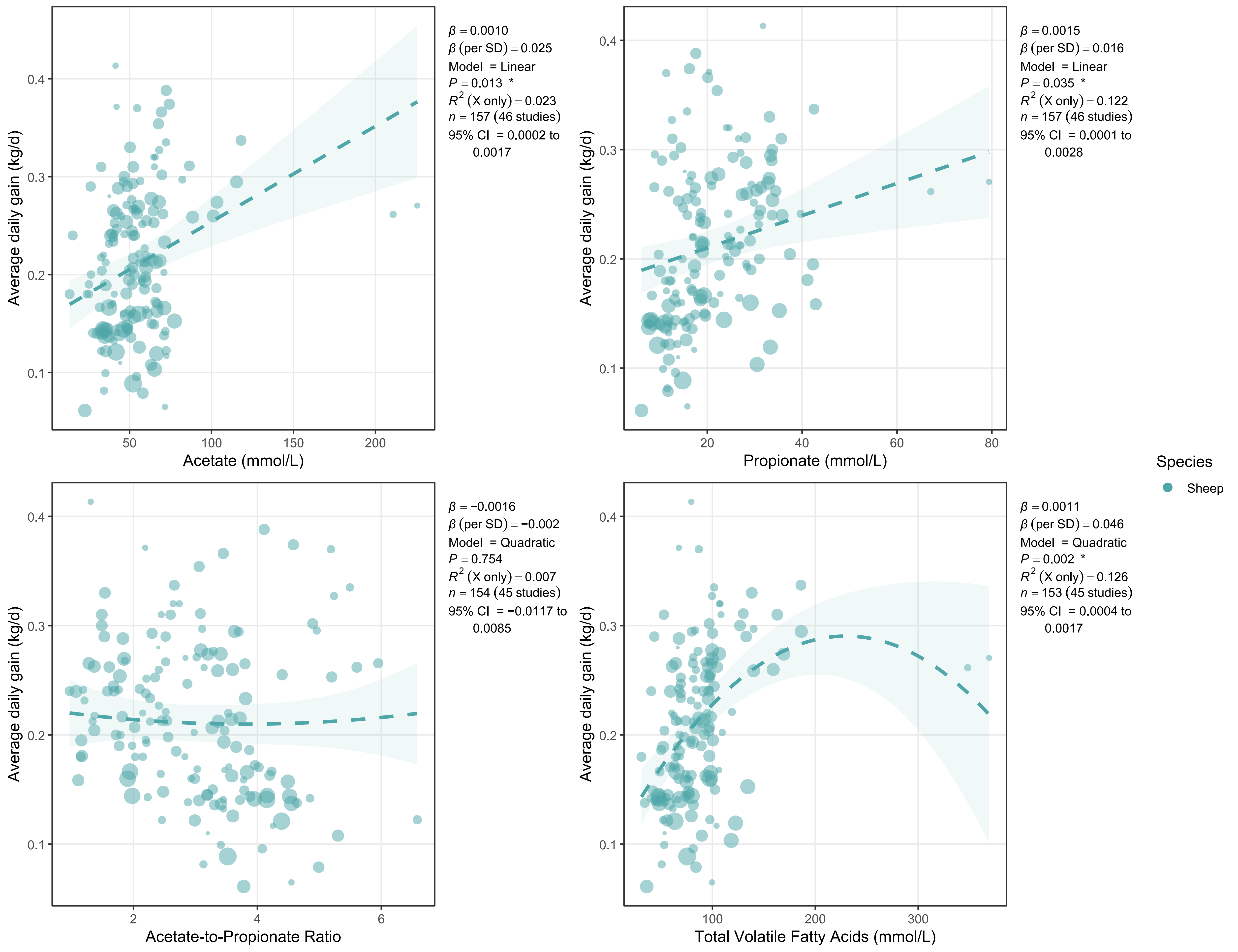

β = 0.0010
β (per SD) = 0.025
Model = Linear
P = 0.013 *
R² (X only) = 0.023
n = 157 (46 studies)
95% CI = 0.0002 to 0.0017
Average daily gain (kg/d)
Acetate (mmol/L)
β = 0.0015
β (per SD) = 0.016
Model = Linear
P = 0.035 *
R² (X only) = 0.122
n = 157 (46 studies)
95% CI = 0.0001 to 0.0028
Propionate (mmol/L)
Species
Sheep
β = −0.0016
β (per SD) = −0.002
Model = Quadratic
P = 0.754
R² (X only) = 0.007
n = 154 (45 studies)
95% CI = −0.0117 to 0.0085
Acetate-to-Propionate Ratio
β = 0.0011
β (per SD) = 0.046
Model = Quadratic
P = 0.002 *
R² (X only) = 0.126
n = 153 (45 studies)
95% CI = 0.0004 to 0.0017
Total Volatile Fatty Acids (mmol/L)
