# Supplementary material for: Association of Ruminal Volatile Fatty Acids with Average Daily Gain in Growing Sheep and Goats: A Systematic Review and Meta-Regression Analysis
Source: Animals (Basel). 2026 Oct 27;16(21):3386. doi: 10.3390/ani16213386 (PMC13761521; doi:10.3390/ani16213386)
Supplement: Supplementary file 1 [file animals-16-03386-s001.zip › S1 Methods and Forest Plots/Figure S3 ADG forest plot (goat).pdf]

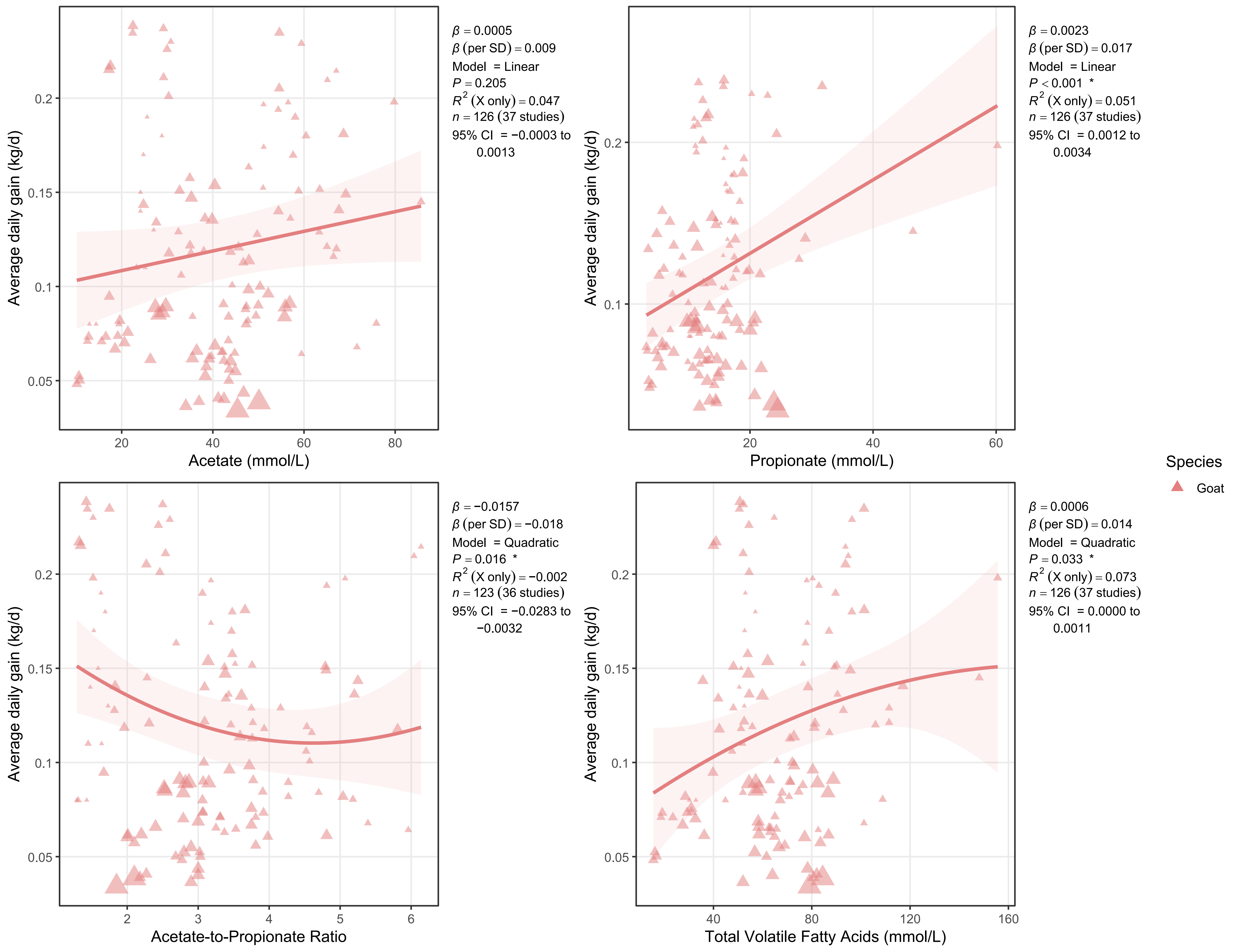
β = 0.0005
β (per SD) = 0.009
Model = Linear
P = 0.205
R^2 (X only) = 0.047
n = 126 (37 studies)
95% CI = −0.0003 to 0.0013
Average daily gain (kg/d)
Acetate (mmol/L)
β = 0.0023
β (per SD) = 0.017
Model = Linear
P < 0.001 *
R^2 (X only) = 0.051
n = 126 (37 studies)
95% CI = 0.0012 to 0.0034
Average daily gain (kg/d)
Propionate (mmol/L)
β = −0.0157
β (per SD) = −0.018
Model = Quadratic
P = 0.016 *
R^2 (X only) = −0.002
n = 123 (36 studies)
95% CI = −0.0283 to −0.0032
Average daily gain (kg/d)
Acetate-to-Propionate Ratio
β = 0.0006
β (per SD) = 0.014
Model = Quadratic
P = 0.033 *
R^2 (X only) = 0.073
n = 126 (37 studies)
95% CI = 0.0000 to 0.0011
Average daily gain (kg/d)
Total Volatile Fatty Acids (mmol/L)
Species
Goat
