# Supplementary material for: Association of Ruminal Volatile Fatty Acids with Average Daily Gain in Growing Sheep and Goats: A Systematic Review and Meta-Regression Analysis
Source: Animals (Basel). 2026 Oct 27;16(21):3386. doi: 10.3390/ani16213386 (PMC13761521; doi:10.3390/ani16213386)
Supplement: Supplementary file 1 [file animals-16-03386-s001.zip › S1 Methods and Forest Plots/Figure S7 Funnel plot (total VFA).pdf]

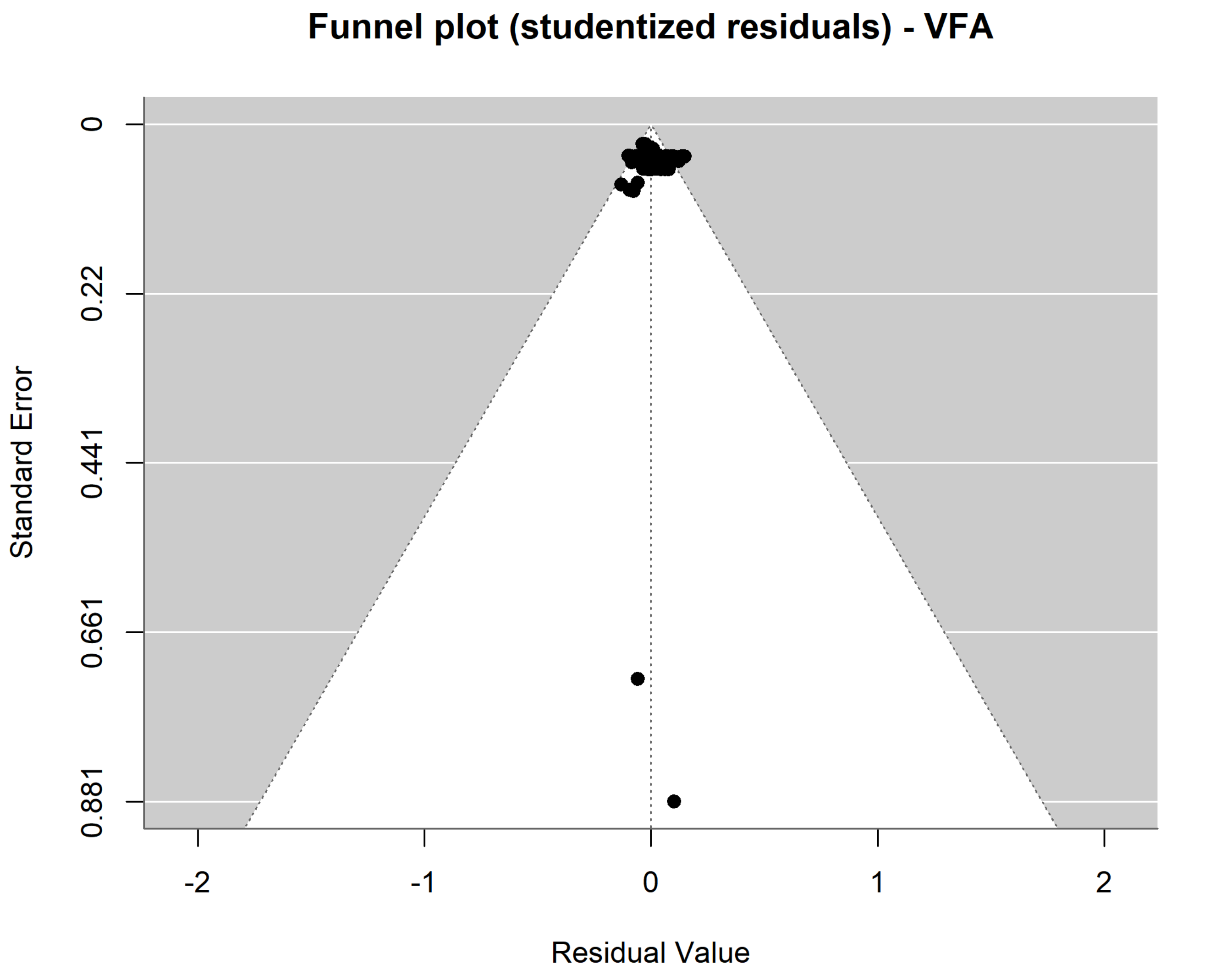
Funnel plot (studentized residuals) - VFA
Standard Error
0
0.22
0.441
0.661
0.881
-2
-1
0
1
2
Residual Value
