# Supplementary material for: Association of Ruminal Volatile Fatty Acids with Average Daily Gain in Growing Sheep and Goats: A Systematic Review and Meta-Regression Analysis
Source: Animals (Basel). 2026 Oct 27;16(21):3386. doi: 10.3390/ani16213386 (PMC13761521; doi:10.3390/ani16213386)
Supplement: Supplementary file 1 [file animals-16-03386-s001.zip › S1 Methods and Forest Plots/Supplementary Text S1 Search and screening log.pdf]

```
====================================================================
SUPPLEMENTARY MATERIAL S1 - SEARCH AND SCREENING LOG (PRISMA flow)
====================================================================
Final counts match the main text (84 unique studies / 290
treatment groups; 82 with complete data / 283 treatment groups;
total VFA: 81 studies / 279 treatment groups; one included
publication contributed both a sheep and a goat arm and was
treated as a single study).
--------------------------------------------------------------------
1. SEARCH DATE AND UPDATES
   - Initial search started: January 2026 (exact day not recorded;
     searches were run manually, keyword by keyword)
   - Search cutoff / last update: 2026-05-20
   Note: no upper date limit was imposed; the search covered all years
   up to the cutoff (included articles published in 2026).
--------------------------------------------------------------------
2. DATABASES / PLATFORMS
   - PubMed
   - Web of Science
   - CNKI and other Chinese databases (e.g., WanFang)
   All searches were performed manually, entering each keyword
   separately and screening results by hand; per-database search
   dates were not logged.
--------------------------------------------------------------------
3. SEARCH STRATEGY (manual keyword-by-keyword searching)
   English keywords: rumen volatile fatty acid(s), acetate /
   acetic acid, propionate / propionic acid, acetate-to-propionate
   ratio (A:P), total VFA; combined with average daily gain (ADG),
   daily gain, growth performance; and sheep, ovine, lamb, goat,
   caprine, kid.
   Chinese keywords: 瘤胃 挥发性脂肪酸 乙酸 丙酸 乙丙比;
   平均日增重 生长性能; 绵羊 山羊 羔羊.
   Each keyword was searched individually in each database, and the
   results were screened manually against the eligibility criteria.
--------------------------------------------------------------------
4. SCREENING PROCESS (PRISMA flow; matches Methods 2.1; cut-off 2026-05-20)
   - Records identified (before deduplication): 1,846
   - After deduplication: 1,275
   - Excluded by title/abstract screening: 1,085
   - Full-text articles assessed for eligibility: 190
   - Full-text articles excluded (total 105). Exclusion reasons
     (non-exclusive; applied during manual screening):
       Not sheep or goats
       ADG or VFA metrics not reported
       Missing SD or SE for computing group-level effect data
       Duplicate publication / data unusable
     Note: because screening was performed manually keyword by
     keyword, a mutually exclusive count per reason was not tracked;
     the four reason categories above are reported as in Methods 2.1
     ("excluded due to reasons such as ...").
   - INCLUDED: 84 unique studies / 290 treatment groups
       (48 studies contributed sheep groups / 164 groups; 37 studies
       contributed goat groups / 126 groups; one study contributed
       both species)
   - Complete-case sample: 82 unique studies / 283 treatment groups
       (46 studies contributed sheep groups / 157 groups; 37 studies
       contributed goat groups / 126 groups)
   - Total VFA analysis: 81 studies / 279 treatment groups
--------------------------------------------------------------------
5. DATA EXTRACTION AND QUALITY CHECK
   - Two researchers independently extracted data from the original
     manuscripts of each included study and cross-verified all
     records; missing records were excluded according to the
     complete-data principle [done].
```

----

6. PRISMA 2020 CHECKLIST

- Completed in the file "PRISMA_2020_checklist.docx" (PRISMA 2020 statement template); verify reporting locations at submission.
