# Supplementary material for: Association of Ruminal Volatile Fatty Acids with Average Daily Gain in Growing Sheep and Goats: A Systematic Review and Meta-Regression Analysis
Source: Animals (Basel). 2026 Oct 27;16(21):3386. doi: 10.3390/ani16213386 (PMC13761521; doi:10.3390/ani16213386)
Supplement: Supplementary file 1 [file animals-16-03386-s001.zip › S2 FCR Results/Figure S10 FCR forest plot (goat).pdf]

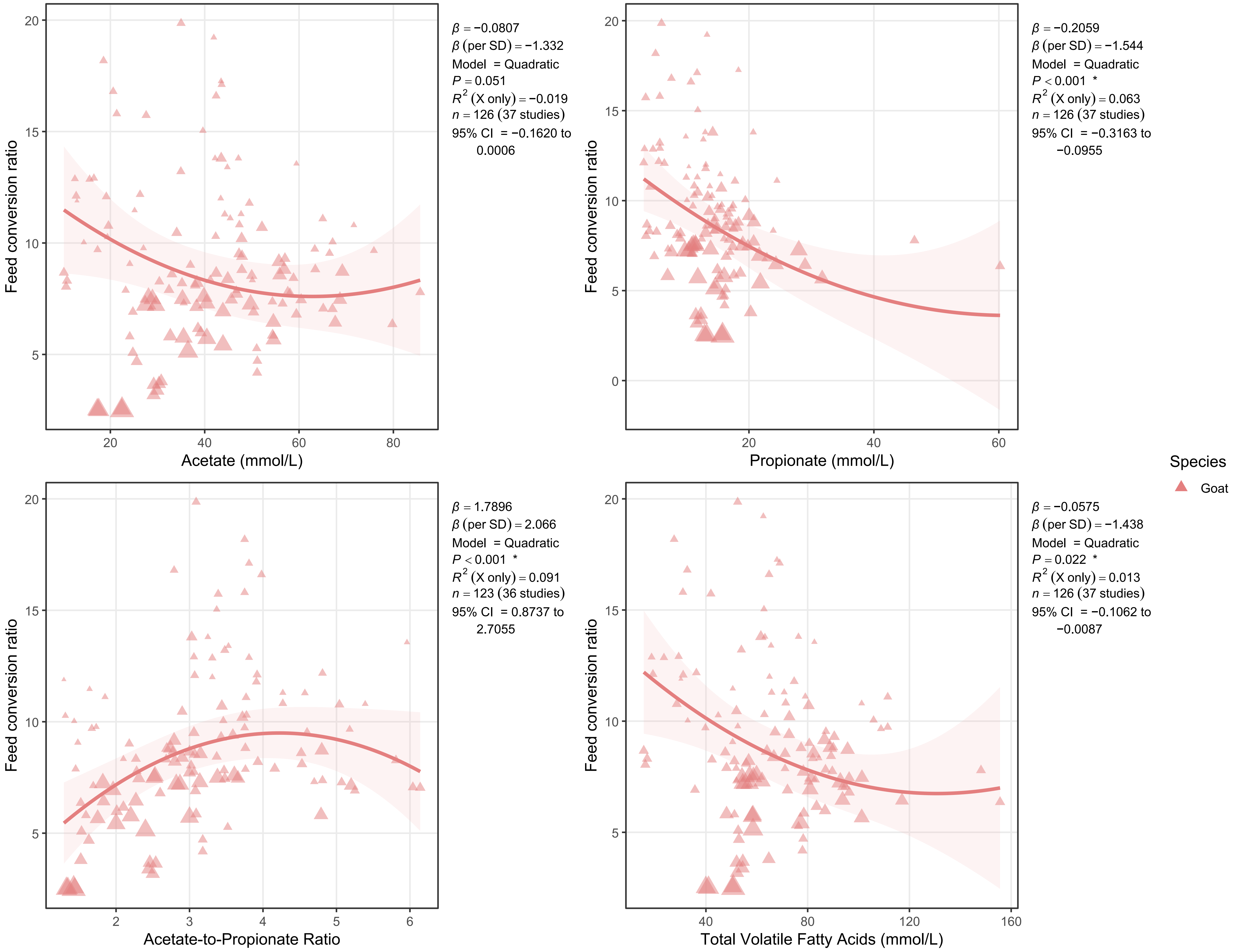
Feed conversion ratio
Acetate (mmol/L)
β = −0.0807
β (per SD) = −1.332
Model = Quadratic
P = 0.051
R2 (X only) = −0.019
n = 126 (37 studies)
95% CI = −0.1620 to 0.0006
Propionate (mmol/L)
β = −0.2059
β (per SD) = −1.544
Model = Quadratic
P < 0.001 *
R2 (X only) = 0.063
n = 126 (37 studies)
95% CI = −0.3163 to −0.0955
Acetate-to-Propionate Ratio
β = 1.7896
β (per SD) = 2.066
Model = Quadratic
P < 0.001 *
R2 (X only) = 0.091
n = 123 (36 studies)
95% CI = 0.8737 to 2.7055
Total Volatile Fatty Acids (mmol/L)
β = −0.0575
β (per SD) = −1.438
Model = Quadratic
P = 0.022 *
R2 (X only) = 0.013
n = 126 (37 studies)
95% CI = −0.1062 to −0.0087
Species
Goat
