# Supplementary material for: Association of Ruminal Volatile Fatty Acids with Average Daily Gain in Growing Sheep and Goats: A Systematic Review and Meta-Regression Analysis
Source: Animals (Basel). 2026 Oct 27;16(21):3386. doi: 10.3390/ani16213386 (PMC13761521; doi:10.3390/ani16213386)
Supplement: Supplementary file 1 [file animals-16-03386-s001.zip › S2 FCR Results/Figure S11 FCR LMM (total).pdf]

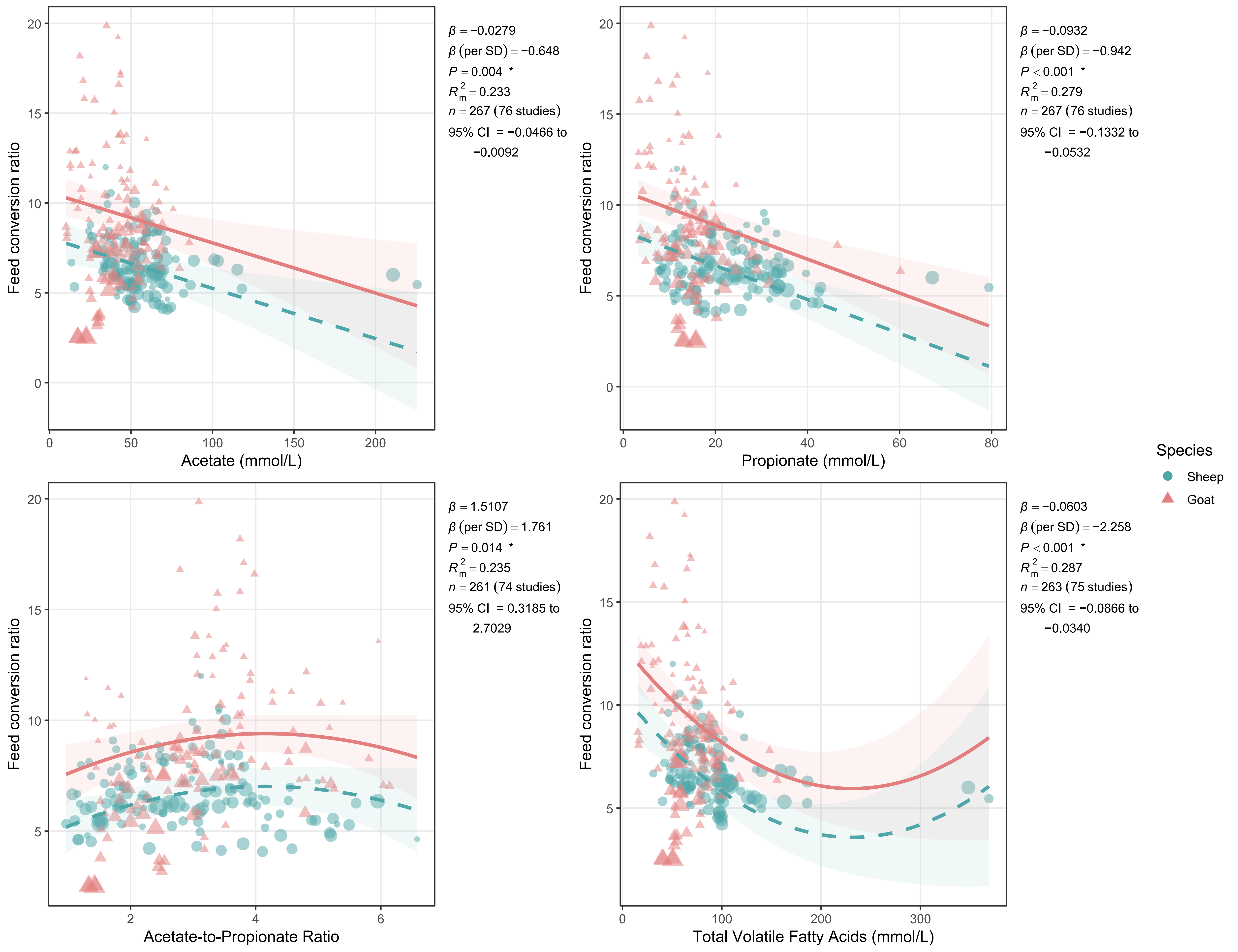
β = −0.0279
β (per SD) = −0.648
P = 0.004 *
R²m = 0.233
n = 267 (76 studies)
95% CI = −0.0466 to −0.0092
Feed conversion ratio
Acetate (mmol/L)
β = −0.0932
β (per SD) = −0.942
P < 0.001 *
R²m = 0.279
n = 267 (76 studies)
95% CI = −0.1332 to −0.0532
Feed conversion ratio
Propionate (mmol/L)
Species
Sheep
Goat
β = 1.5107
β (per SD) = 1.761
P = 0.014 *
R²m = 0.235
n = 261 (74 studies)
95% CI = 0.3185 to 2.7029
Feed conversion ratio
Acetate-to-Propionate Ratio
β = −0.0603
β (per SD) = −2.258
P < 0.001 *
R²m = 0.287
n = 263 (75 studies)
95% CI = −0.0866 to −0.0340
Feed conversion ratio
Total Volatile Fatty Acids (mmol/L)
