# Supplementary material for: Association of Ruminal Volatile Fatty Acids with Average Daily Gain in Growing Sheep and Goats: A Systematic Review and Meta-Regression Analysis
Source: Animals (Basel). 2026 Oct 27;16(21):3386. doi: 10.3390/ani16213386 (PMC13761521; doi:10.3390/ani16213386)
Supplement: Supplementary file 1 [file animals-16-03386-s001.zip › S2 FCR Results/Figure S12 FCR LMM (sheep).pdf]

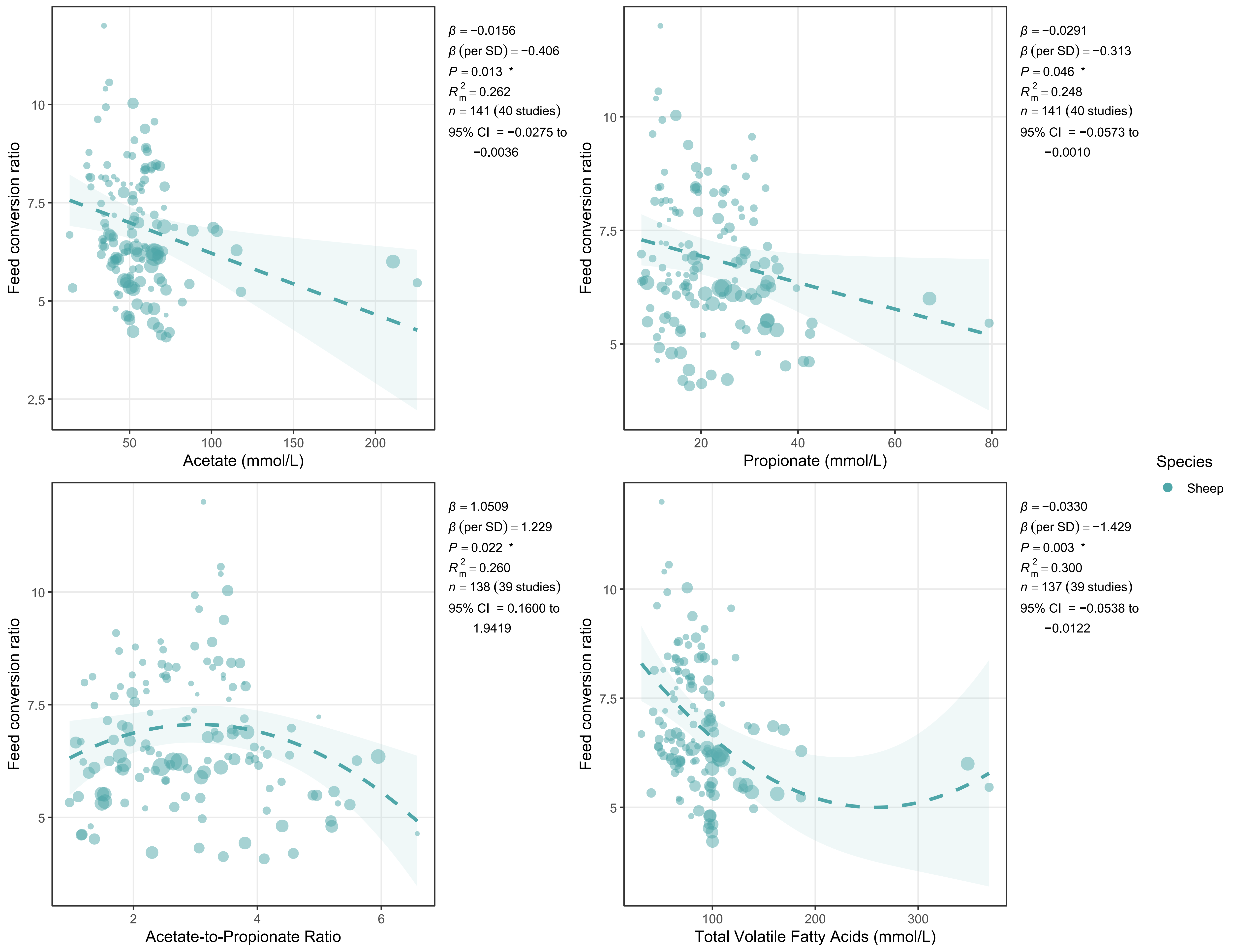
β = −0.0156
β (per SD) = −0.406
P = 0.013 *
R²m = 0.262
n = 141 (40 studies)
95% CI = −0.0275 to −0.0036
Feed conversion ratio
Acetate (mmol/L)
β = −0.0291
β (per SD) = −0.313
P = 0.046 *
R²m = 0.248
n = 141 (40 studies)
95% CI = −0.0573 to −0.0010
Feed conversion ratio
Propionate (mmol/L)
Species
Sheep
β = 1.0509
β (per SD) = 1.229
P = 0.022 *
R²m = 0.260
n = 138 (39 studies)
95% CI = 0.1600 to 1.9419
Feed conversion ratio
Acetate-to-Propionate Ratio
β = −0.0330
β (per SD) = −1.429
P = 0.003 *
R²m = 0.300
n = 137 (39 studies)
95% CI = −0.0538 to −0.0122
Feed conversion ratio
Total Volatile Fatty Acids (mmol/L)
