# Supplementary material for: Association of Ruminal Volatile Fatty Acids with Average Daily Gain in Growing Sheep and Goats: A Systematic Review and Meta-Regression Analysis
Source: Animals (Basel). 2026 Oct 27;16(21):3386. doi: 10.3390/ani16213386 (PMC13761521; doi:10.3390/ani16213386)
Supplement: Supplementary file 1 [file animals-16-03386-s001.zip › S2 FCR Results/Figure S13 FCR LMM (goat).pdf]

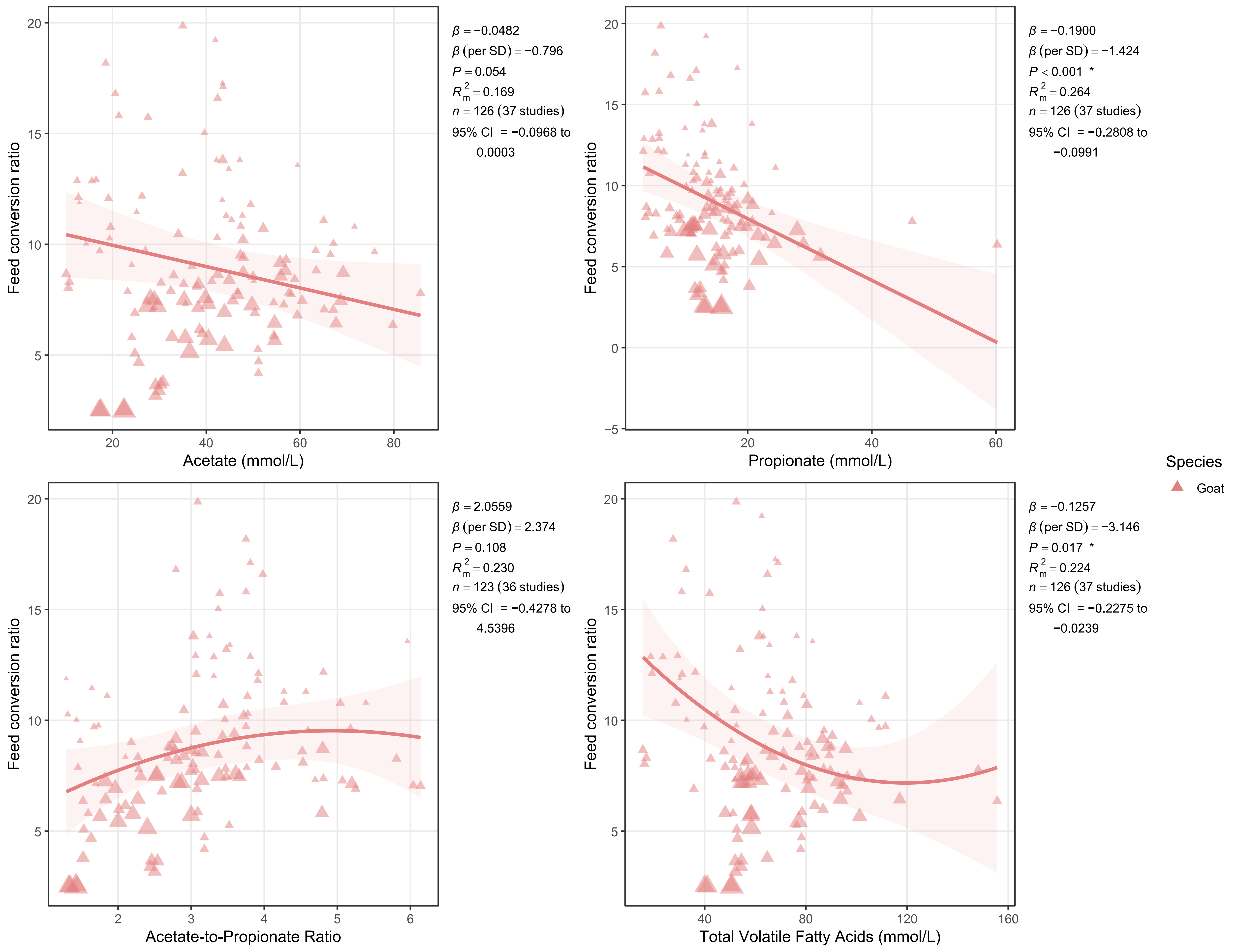
β = −0.0482
β (per SD) = −0.796
P = 0.054
R²m = 0.169
n = 126 (37 studies)
95% CI = −0.0968 to 0.0003
Feed conversion ratio
Acetate (mmol/L)
β = −0.1900
β (per SD) = −1.424
P < 0.001 *
R²m = 0.264
n = 126 (37 studies)
95% CI = −0.2808 to −0.0991
Propionate (mmol/L)
Species
Goat
β = 2.0559
β (per SD) = 2.374
P = 0.108
R²m = 0.230
n = 123 (36 studies)
95% CI = −0.4278 to 4.5396
Acetate-to-Propionate Ratio
β = −0.1257
β (per SD) = −3.146
P = 0.017 *
R²m = 0.224
n = 126 (37 studies)
95% CI = −0.2275 to −0.0239
Total Volatile Fatty Acids (mmol/L)
