# Supplementary material for: Association of Ruminal Volatile Fatty Acids with Average Daily Gain in Growing Sheep and Goats: A Systematic Review and Meta-Regression Analysis
Source: Animals (Basel). 2026 Oct 27;16(21):3386. doi: 10.3390/ani16213386 (PMC13761521; doi:10.3390/ani16213386)
Supplement: Supplementary file 1 [file animals-16-03386-s001.zip › S2 FCR Results/Figure S14 FCR incremental CV-R2.pdf]

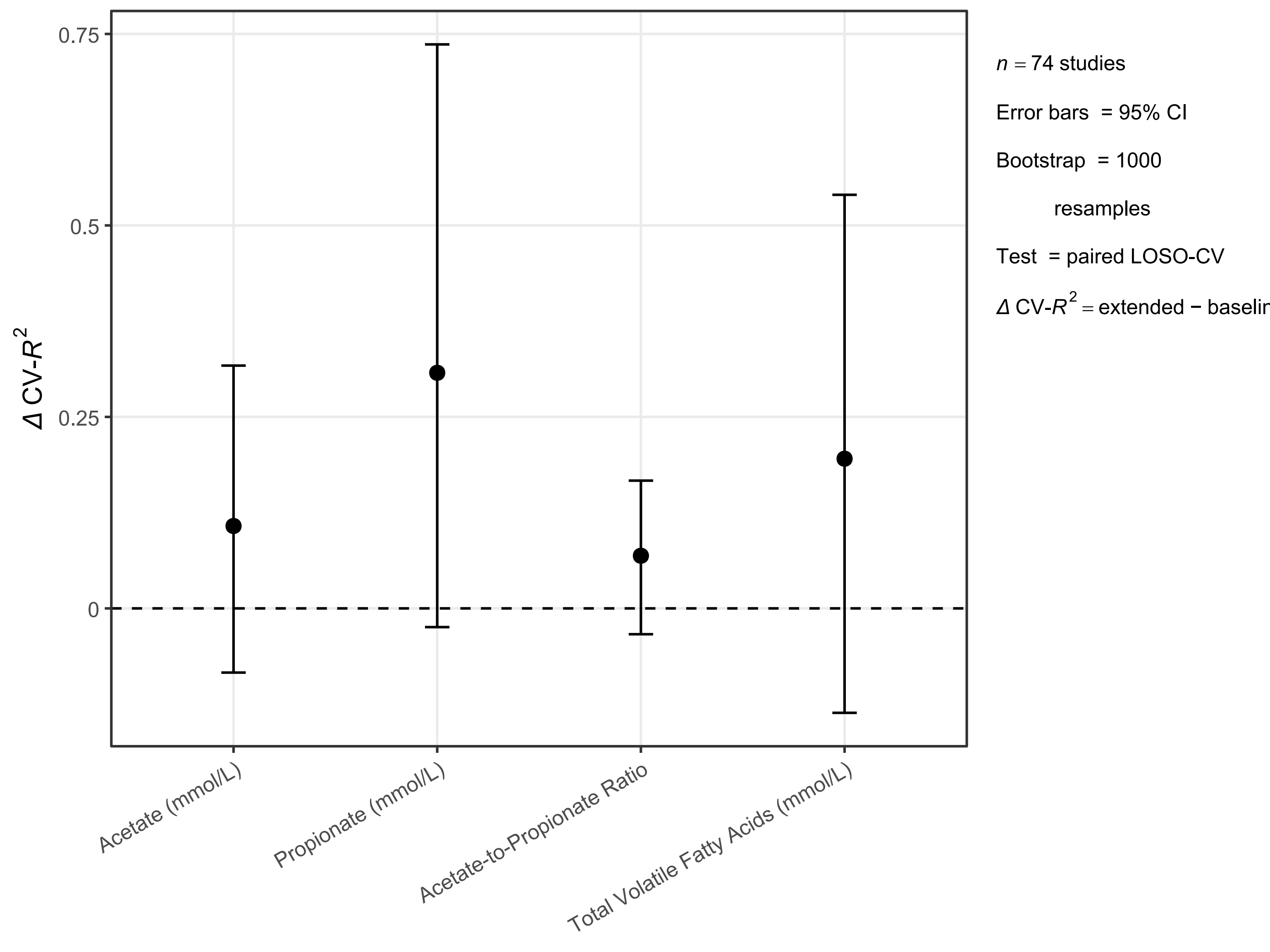

0.75
0.5
0.25
0
Δ CV-R²
Acetate (mmol/L)
Propionate (mmol/L)
Acetate-to-Propionate Ratio
Total Volatile Fatty Acids (mmol/L)
n = 74 studies
Error bars = 95% CI
Bootstrap = 1000
resamples
Test = paired LOSO-CV
Δ CV-R² = extended − baselin
