# Supplementary material for: Association of Ruminal Volatile Fatty Acids with Average Daily Gain in Growing Sheep and Goats: A Systematic Review and Meta-Regression Analysis
Source: Animals (Basel). 2026 Oct 27;16(21):3386. doi: 10.3390/ani16213386 (PMC13761521; doi:10.3390/ani16213386)
Supplement: Supplementary file 1 [file animals-16-03386-s001.zip › S2 FCR Results/Figure S16 FCR LOSO total-overall (propionate).pdf]

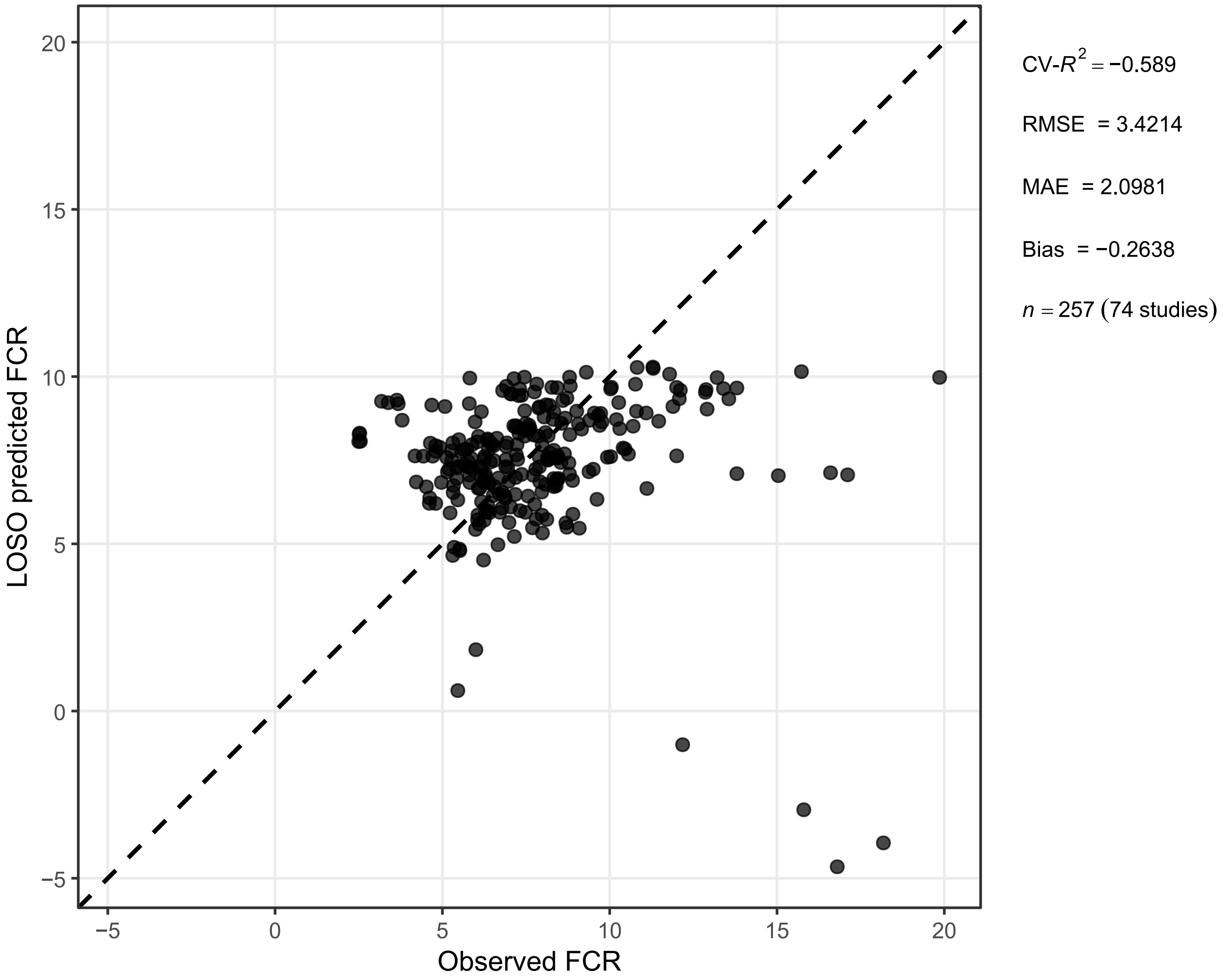
CV-R^2 = −0.589
RMSE = 3.4214
MAE = 2.0981
Bias = −0.2638
n = 257 (74 studies)
LOSO predicted FCR
Observed FCR
20
15
10
5
0
−5
