# Supplementary material for: Association of Ruminal Volatile Fatty Acids with Average Daily Gain in Growing Sheep and Goats: A Systematic Review and Meta-Regression Analysis
Source: Animals (Basel). 2026 Oct 27;16(21):3386. doi: 10.3390/ani16213386 (PMC13761521; doi:10.3390/ani16213386)
Supplement: Supplementary file 1 [file animals-16-03386-s001.zip › S2 FCR Results/Figure S17 FCR LOSO total-sheep (acetate).pdf]

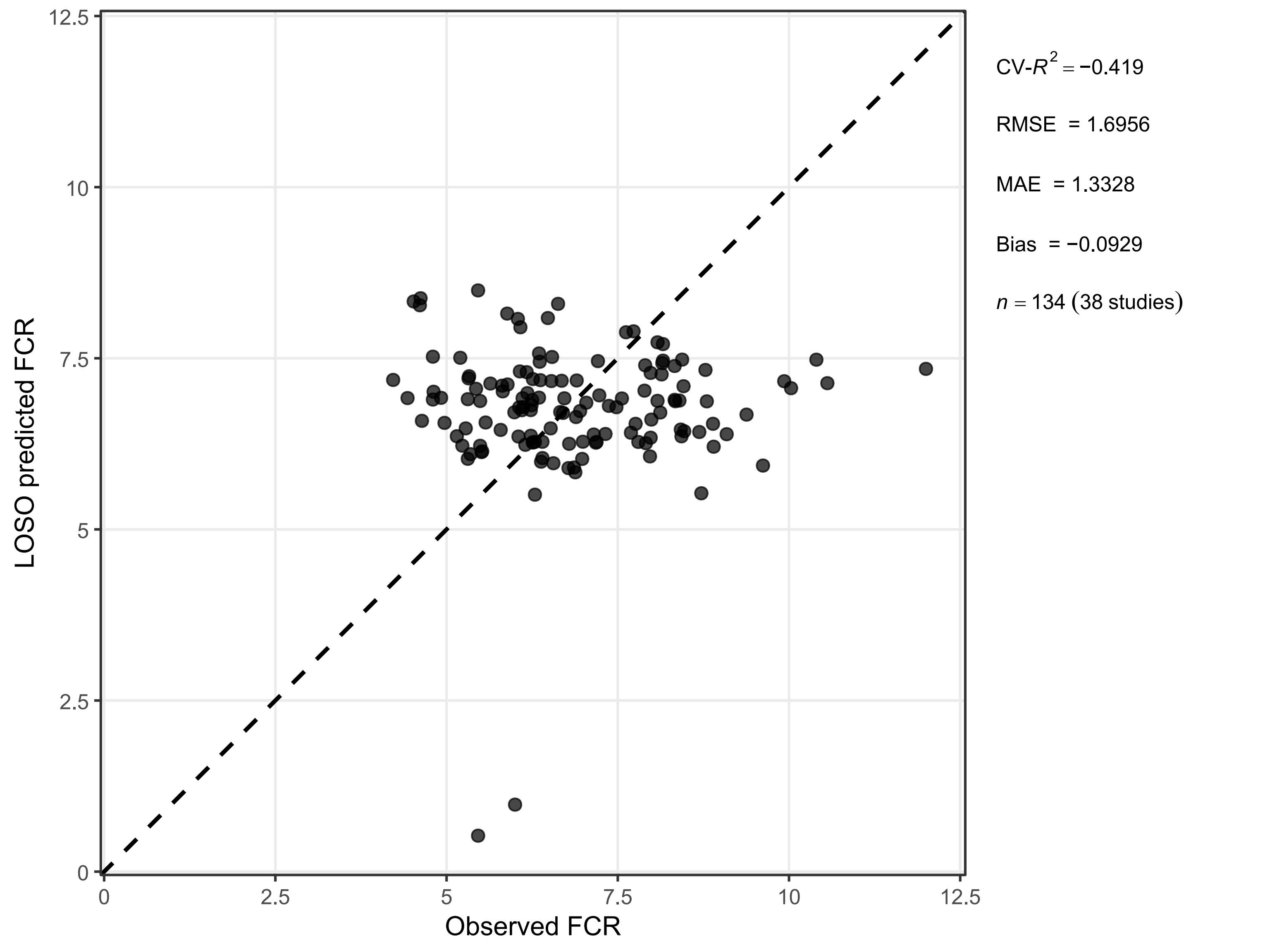

12.5
10
7.5
5
2.5
0
0
2.5
5
7.5
10
12.5
LOSO predicted FCR
Observed FCR
CV-R^2 = −0.419
RMSE = 1.6956
MAE = 1.3328
Bias = −0.0929
n = 134 (38 studies)
