# Supplementary material for: Association of Ruminal Volatile Fatty Acids with Average Daily Gain in Growing Sheep and Goats: A Systematic Review and Meta-Regression Analysis
Source: Animals (Basel). 2026 Oct 27;16(21):3386. doi: 10.3390/ani16213386 (PMC13761521; doi:10.3390/ani16213386)
Supplement: Supplementary file 1 [file animals-16-03386-s001.zip › S2 FCR Results/Figure S18 FCR LOSO total-sheep (A-P ratio).pdf]

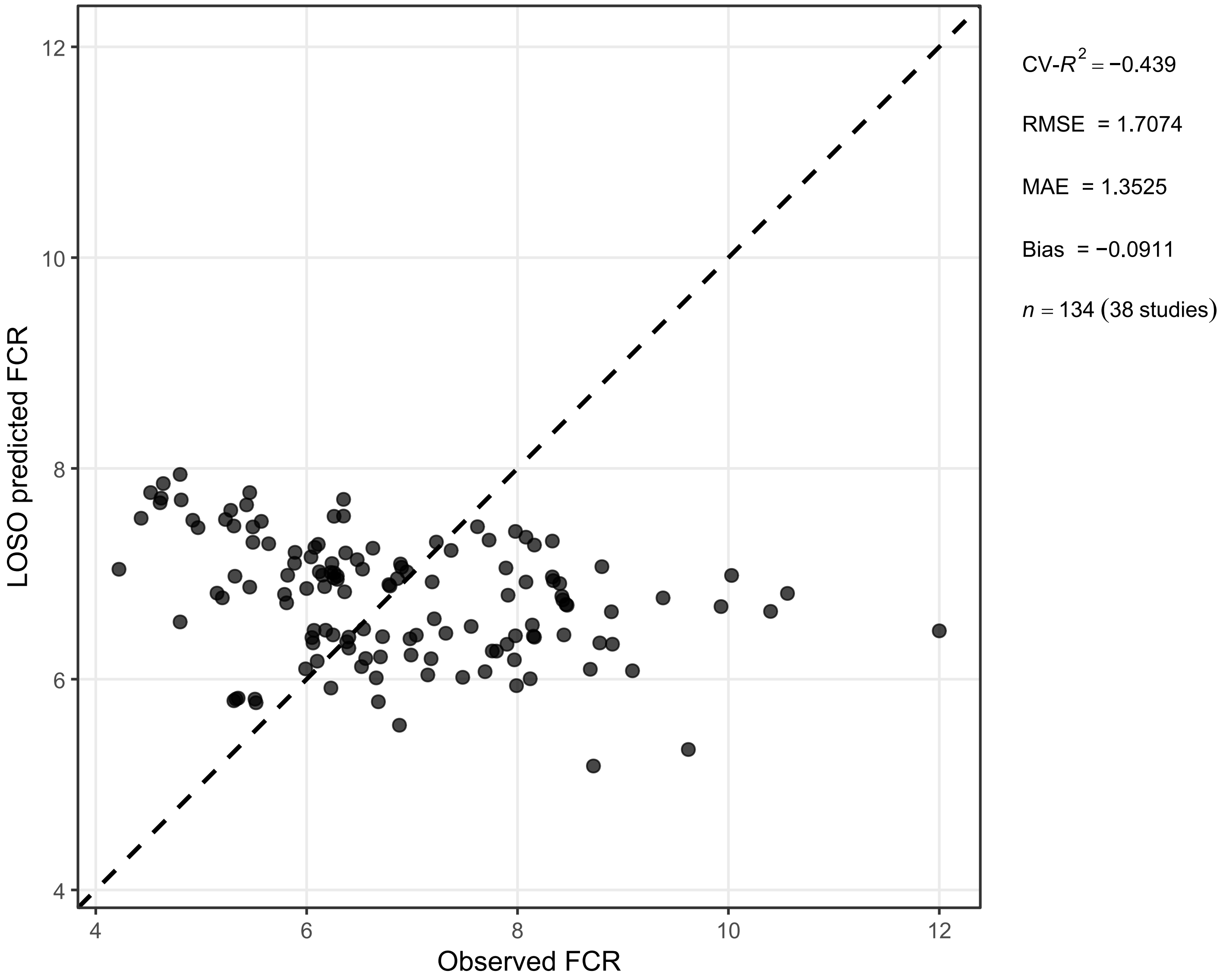

CV-R^2 = −0.439
RMSE = 1.7074
MAE = 1.3525
Bias = −0.0911
n = 134 (38 studies)
LOSO predicted FCR
Observed FCR
4
6
8
10
12
