# Supplementary material for: Association of Ruminal Volatile Fatty Acids with Average Daily Gain in Growing Sheep and Goats: A Systematic Review and Meta-Regression Analysis
Source: Animals (Basel). 2026 Oct 27;16(21):3386. doi: 10.3390/ani16213386 (PMC13761521; doi:10.3390/ani16213386)
Supplement: Supplementary file 1 [file animals-16-03386-s001.zip › S2 FCR Results/Figure S22 FCR LOSO goat-goat (A-P ratio).pdf]

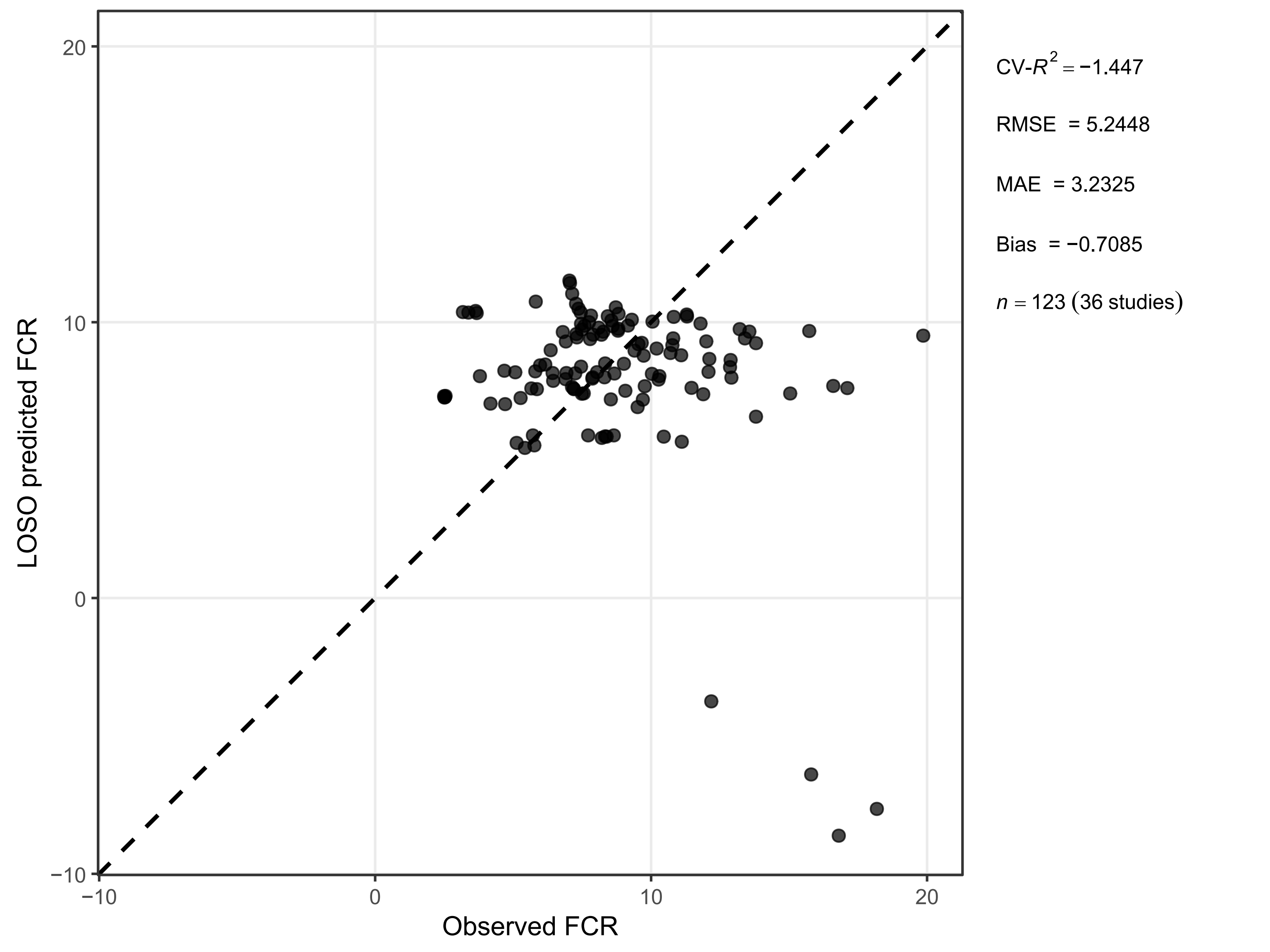

LOSO predicted FCR
Observed FCR
20
10
0
−10
−10
0
10
20
CV-R^2 = −1.447
RMSE = 5.2448
MAE = 3.2325
Bias = −0.7085
n = 123 (36 studies)
