# Supplementary material for: Association of Ruminal Volatile Fatty Acids with Average Daily Gain in Growing Sheep and Goats: A Systematic Review and Meta-Regression Analysis
Source: Animals (Basel). 2026 Oct 27;16(21):3386. doi: 10.3390/ani16213386 (PMC13761521; doi:10.3390/ani16213386)
Supplement: Supplementary file 1 [file animals-16-03386-s001.zip › S2 FCR Results/Figure S23 FCR LOSO goat-goat (propionate).pdf]

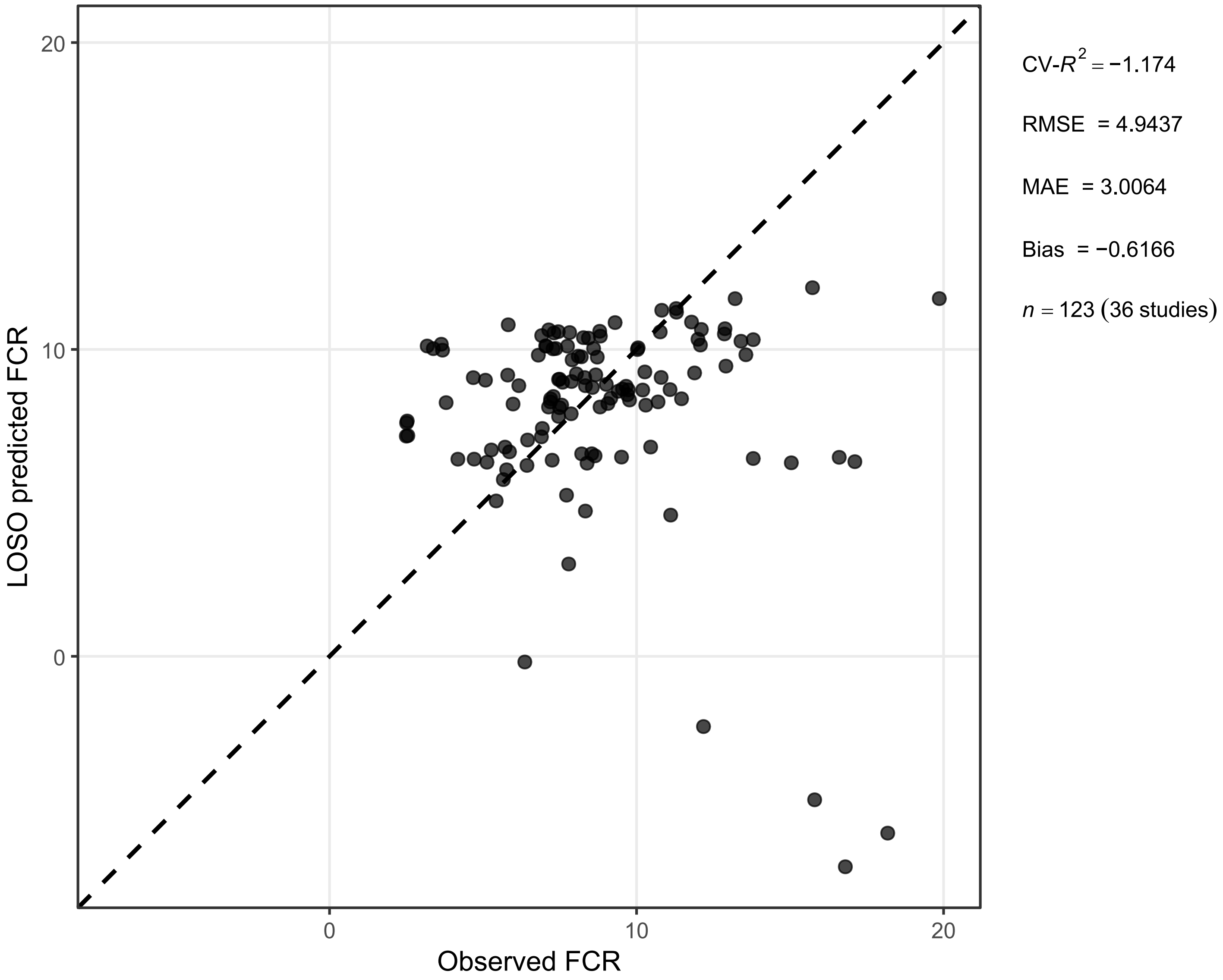

20
10
0
0
10
20
LOSO predicted FCR
Observed FCR
CV-R^2 = −1.174
RMSE = 4.9437
MAE = 3.0064
Bias = −0.6166
n = 123 (36 studies)
