# Supplementary material for: Association of Ruminal Volatile Fatty Acids with Average Daily Gain in Growing Sheep and Goats: A Systematic Review and Meta-Regression Analysis
Source: Animals (Basel). 2026 Oct 27;16(21):3386. doi: 10.3390/ani16213386 (PMC13761521; doi:10.3390/ani16213386)
Supplement: Supplementary file 1 [file animals-16-03386-s001.zip › S2 FCR Results/Figure S8 FCR forest plot (total).pdf]

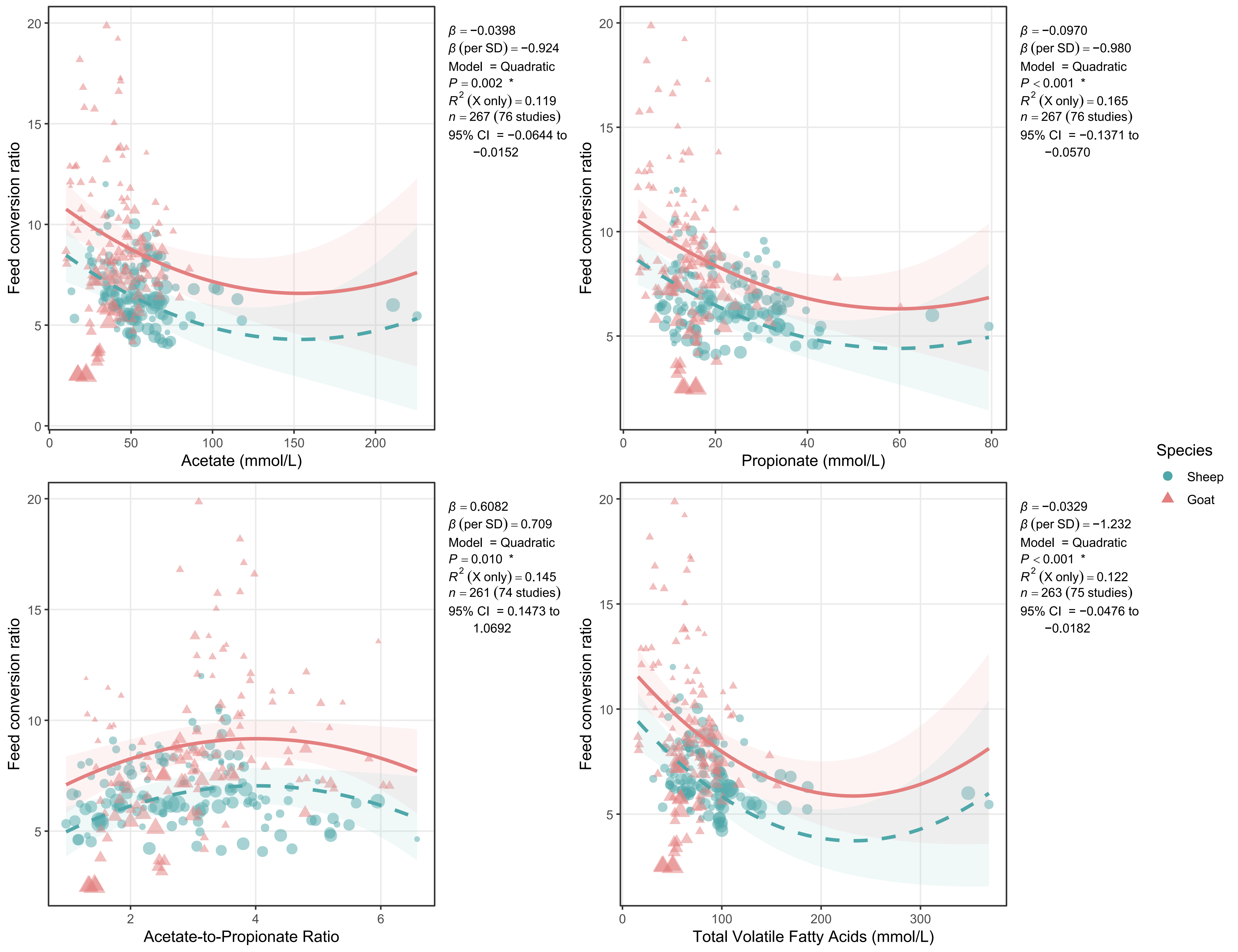

Feed conversion ratio
Acetate (mmol/L)
β = −0.0398
β (per SD) = −0.924
Model = Quadratic
P = 0.002 *
R2 (X only) = 0.119
n = 267 (76 studies)
95% CI = −0.0644 to −0.0152
Propionate (mmol/L)
β = −0.0970
β (per SD) = −0.980
Model = Quadratic
P < 0.001 *
R2 (X only) = 0.165
n = 267 (76 studies)
95% CI = −0.1371 to −0.0570
Species
Sheep
Goat
Acetate-to-Propionate Ratio
β = 0.6082
β (per SD) = 0.709
Model = Quadratic
P = 0.010 *
R2 (X only) = 0.145
n = 261 (74 studies)
95% CI = 0.1473 to 1.0692
Total Volatile Fatty Acids (mmol/L)
β = −0.0329
β (per SD) = −1.232
Model = Quadratic
P < 0.001 *
R2 (X only) = 0.122
n = 263 (75 studies)
95% CI = −0.0476 to −0.0182
