# Supplementary material for: Association of Ruminal Volatile Fatty Acids with Average Daily Gain in Growing Sheep and Goats: A Systematic Review and Meta-Regression Analysis
Source: Animals (Basel). 2026 Oct 27;16(21):3386. doi: 10.3390/ani16213386 (PMC13761521; doi:10.3390/ani16213386)
Supplement: Supplementary file 1 [file animals-16-03386-s001.zip › S2 FCR Results/Figure S9 FCR forest plot (sheep).pdf]

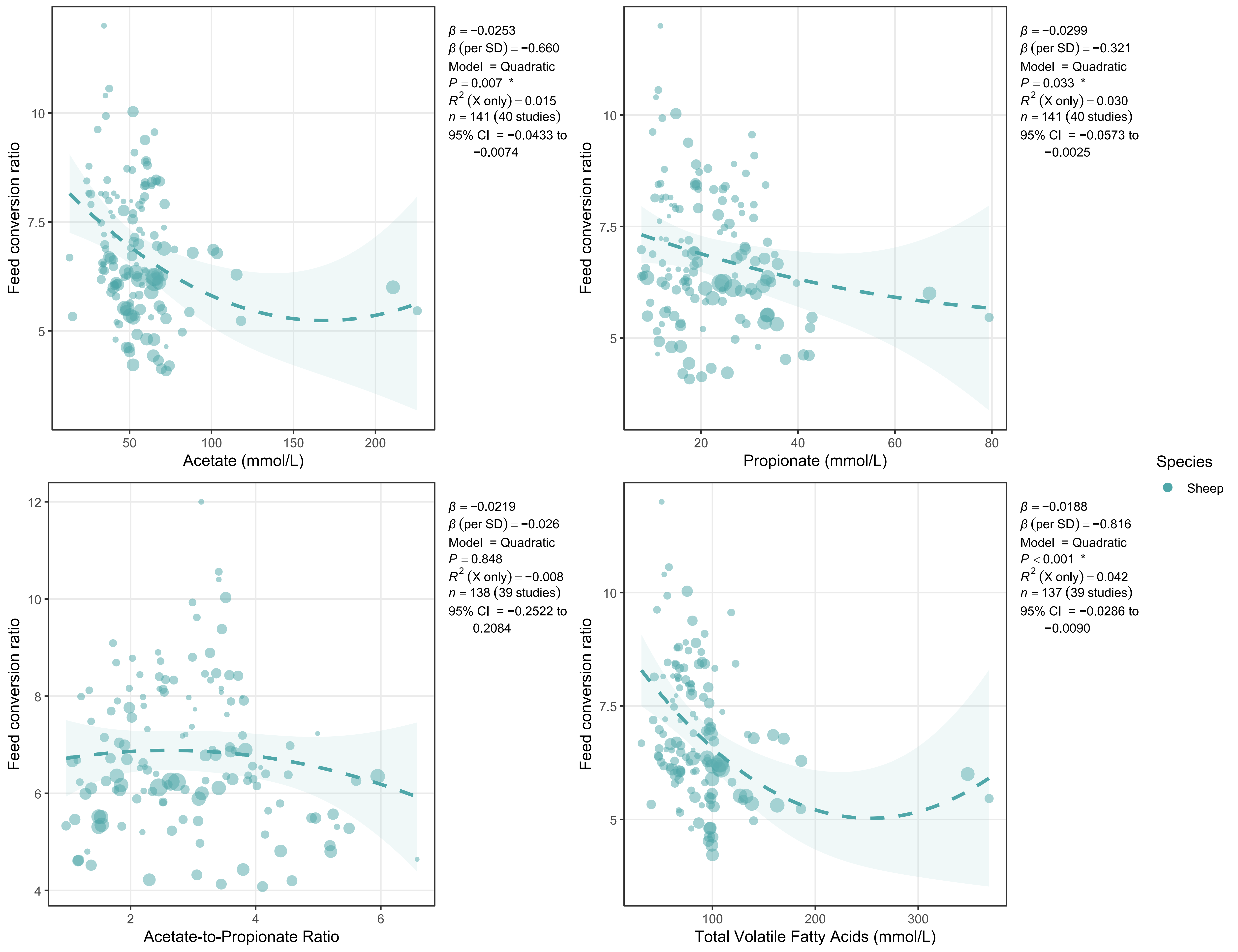

β = −0.0253
β (per SD) = −0.660
Model = Quadratic
P = 0.007 *
R² (X only) = 0.015
n = 141 (40 studies)
95% CI = −0.0433 to −0.0074
Feed conversion ratio
Acetate (mmol/L)
β = −0.0299
β (per SD) = −0.321
Model = Quadratic
P = 0.033 *
R² (X only) = 0.030
n = 141 (40 studies)
95% CI = −0.0573 to −0.0025
Feed conversion ratio
Propionate (mmol/L)
Species
Sheep
β = −0.0219
β (per SD) = −0.026
Model = Quadratic
P = 0.848
R² (X only) = −0.008
n = 138 (39 studies)
95% CI = −0.2522 to 0.2084
Feed conversion ratio
Acetate-to-Propionate Ratio
β = −0.0188
β (per SD) = −0.816
Model = Quadratic
P < 0.001 *
R² (X only) = 0.042
n = 137 (39 studies)
95% CI = −0.0286 to −0.0090
Feed conversion ratio
Total Volatile Fatty Acids (mmol/L)
