# Supplementary material for: Association of Ruminal Volatile Fatty Acids with Average Daily Gain in Growing Sheep and Goats: A Systematic Review and Meta-Regression Analysis
Source: Animals (Basel). 2026 Oct 27;16(21):3386. doi: 10.3390/ani16213386 (PMC13761521; doi:10.3390/ani16213386)
Supplement: Supplementary file 1 [file animals-16-03386-s001.zip › S3 FCR LeaveOneOut/Figure S24 FCR incremental CV-R2 (LOSO).pdf]

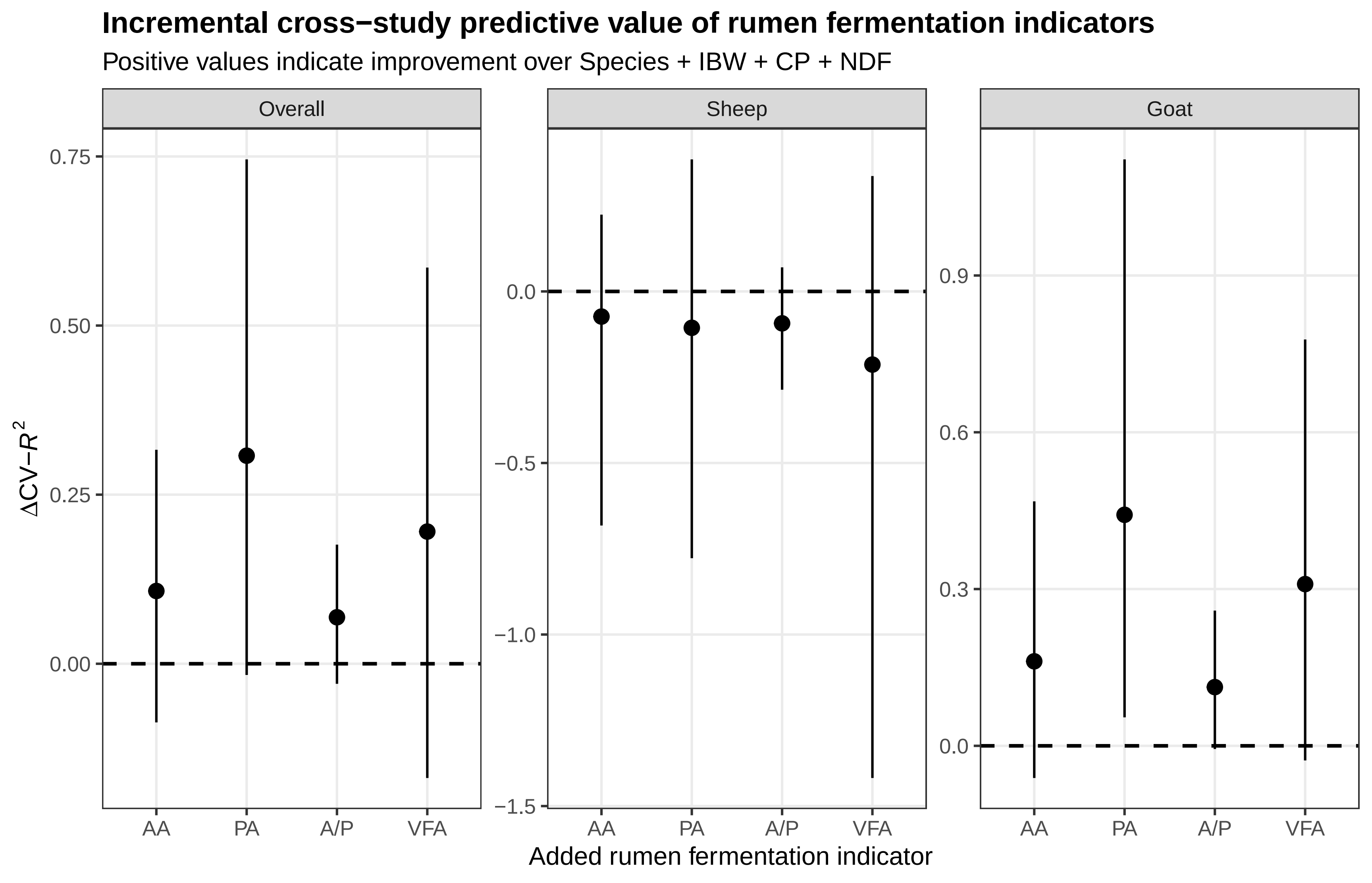

Incremental cross−study predictive value of rumen fermentation indicators
Positive values indicate improvement over Species + IBW + CP + NDF
Overall
Sheep
Goat
ΔCV−R^2
0.75
0.50
0.25
0.00
0.0
−0.5
−1.0
−1.5
0.9
0.6
0.3
0.0
AA
PA
A/P
VFA
Added rumen fermentation indicator
