# Supplementary material for: Association of Ruminal Volatile Fatty Acids with Average Daily Gain in Growing Sheep and Goats: A Systematic Review and Meta-Regression Analysis
Source: Animals (Basel). 2026 Oct 27;16(21):3386. doi: 10.3390/ani16213386 (PMC13761521; doi:10.3390/ani16213386)
Supplement: Supplementary file 1 [file animals-16-03386-s001.zip › S4 FCR Species Interaction/Figure S26 FCR species interaction (propionate, within).pdf]

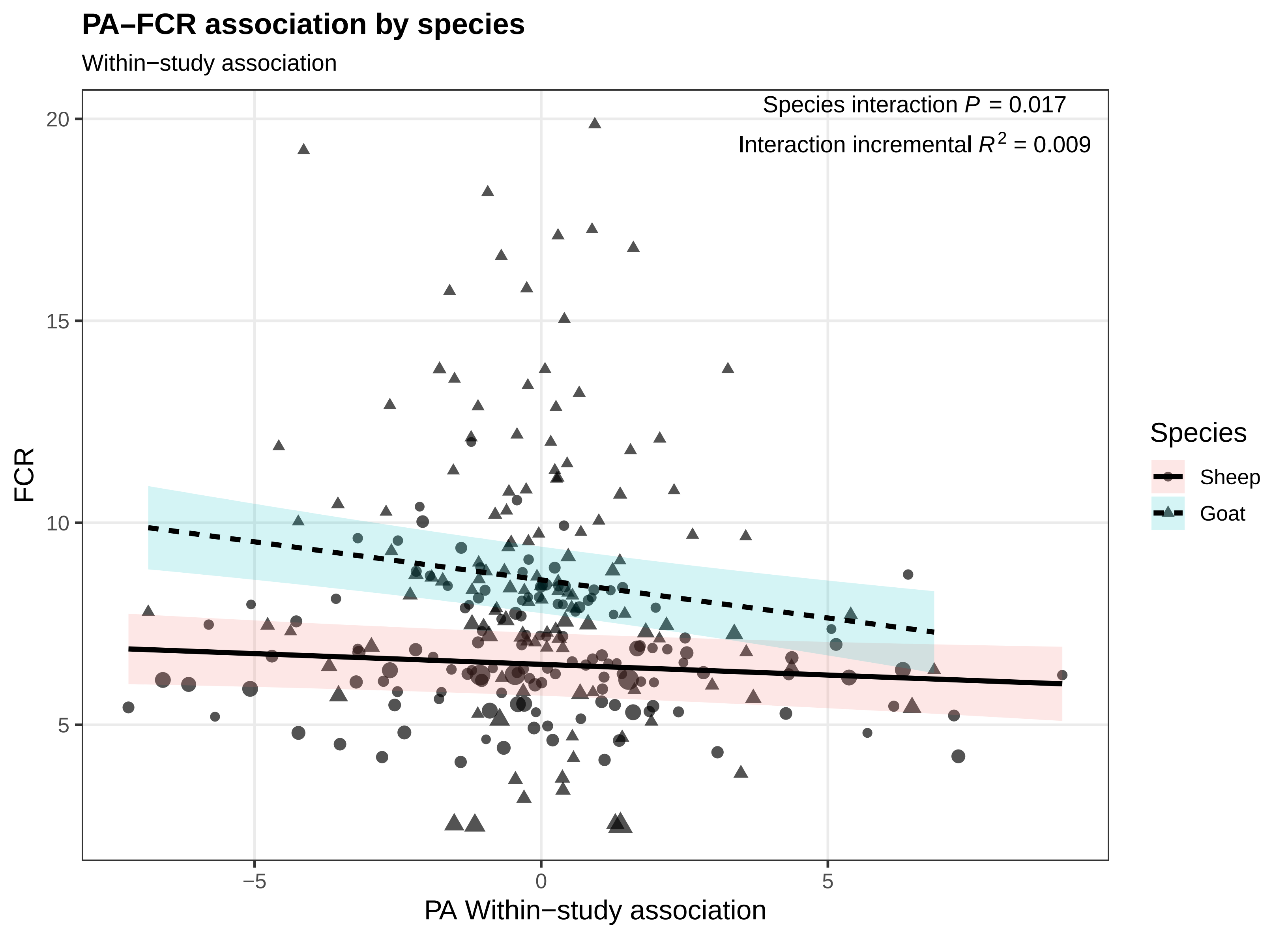
PA−FCR association by species
Within−study association
Species interaction P = 0.017
Interaction incremental R^2 = 0.009
FCR
20
15
10
5
−5
0
5
PA Within−study association
Species
Sheep
Goat
